# Supplementary material for: ADuLT: An efficient and robust time-to-event GWAS
Source: Nat Commun. 2023 Sep 9;14:5553. doi: 10.1038/s41467-023-41210-z (PMC10492844; doi:10.1038/s41467-023-41210-z)
Supplement: Supplementary file 1 — Supplementary Information [file 41467_2023_41210_MOESM1_ESM.pdf]

Supplementary Figures

Simulation Results

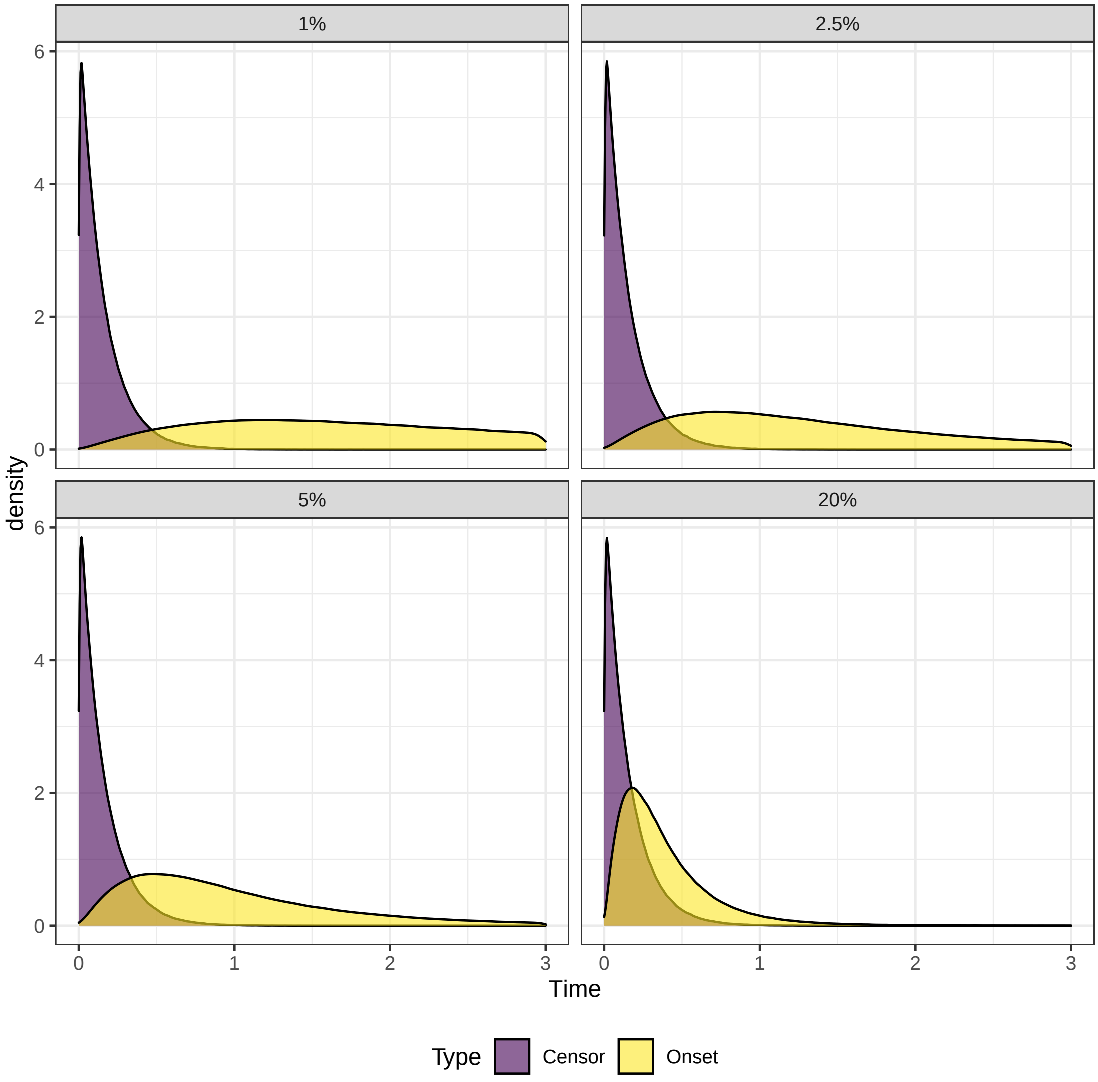

Supplementary Figure 1: **Simulated censoring and age-of-onset times for varying prevalences.** For all 1 million simulated individuals, a censoring, c, and onset $\tilde{t}$, is simulated. For a sub-sample of $20,000$, we illustrate both here for different prevalences. Here, `Censor` refers to the censoring time in all individuals and `Onset` refers to the onset times. An individual is only a case if the onset time occurs before the censoring time.

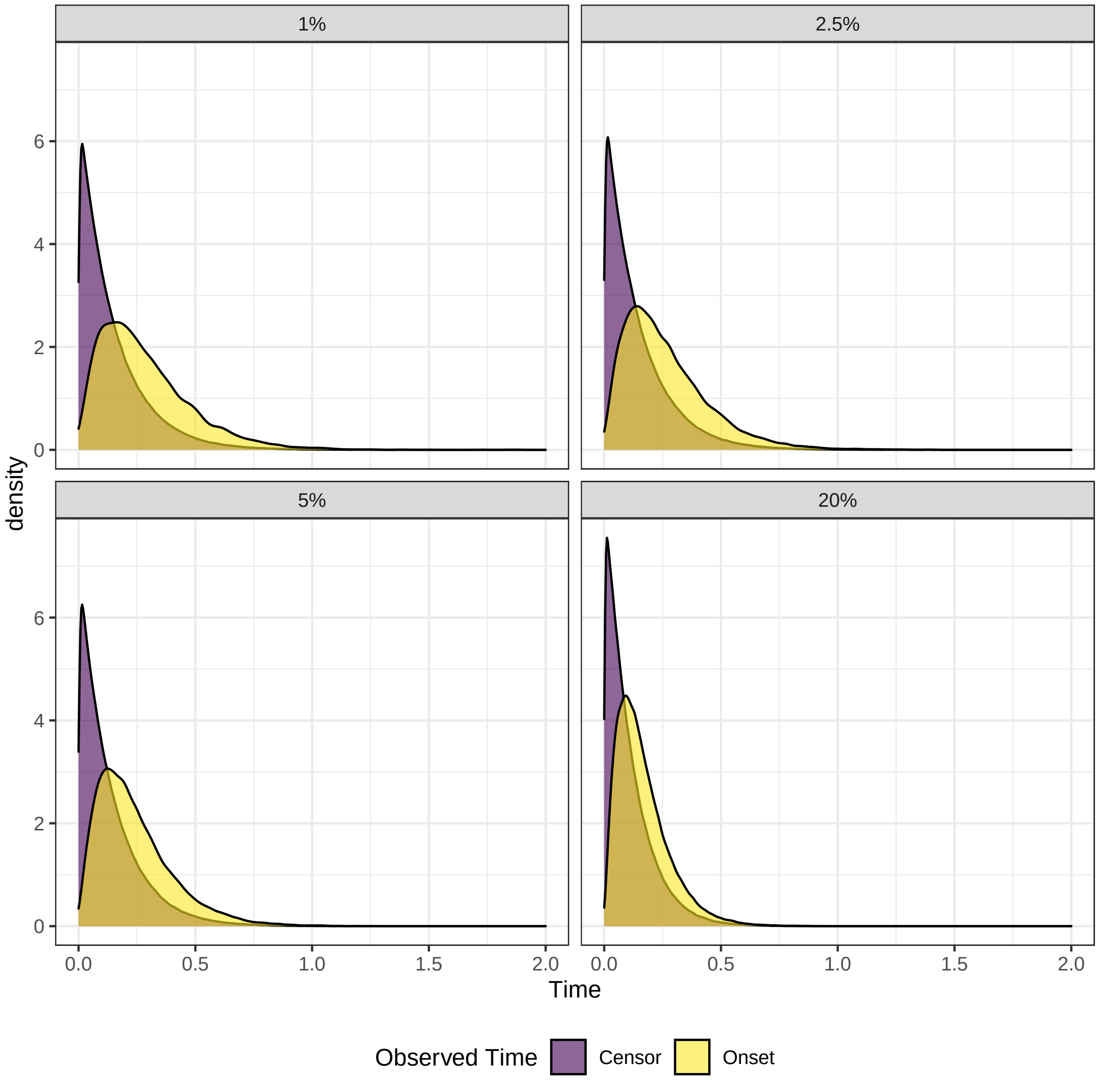

Supplementary Figure 2: **Observed censoring and age-of-onset times in simulations for varying prevalences.** For all 1 million simulated individuals, a censoring, c, and onset $\tilde{t}$, is simulated. For a sub-sample of $20,000$, we illustrate the observed of both here for different prevalences. From these, an observed time is derived as $t = \min(c, \tilde{t})$. The observed onset and censoring times are illustrated for different prevalences. Here, `Censor` refers to the observed censoring time in all controls and `Onset` refers to the onset times for all cases.

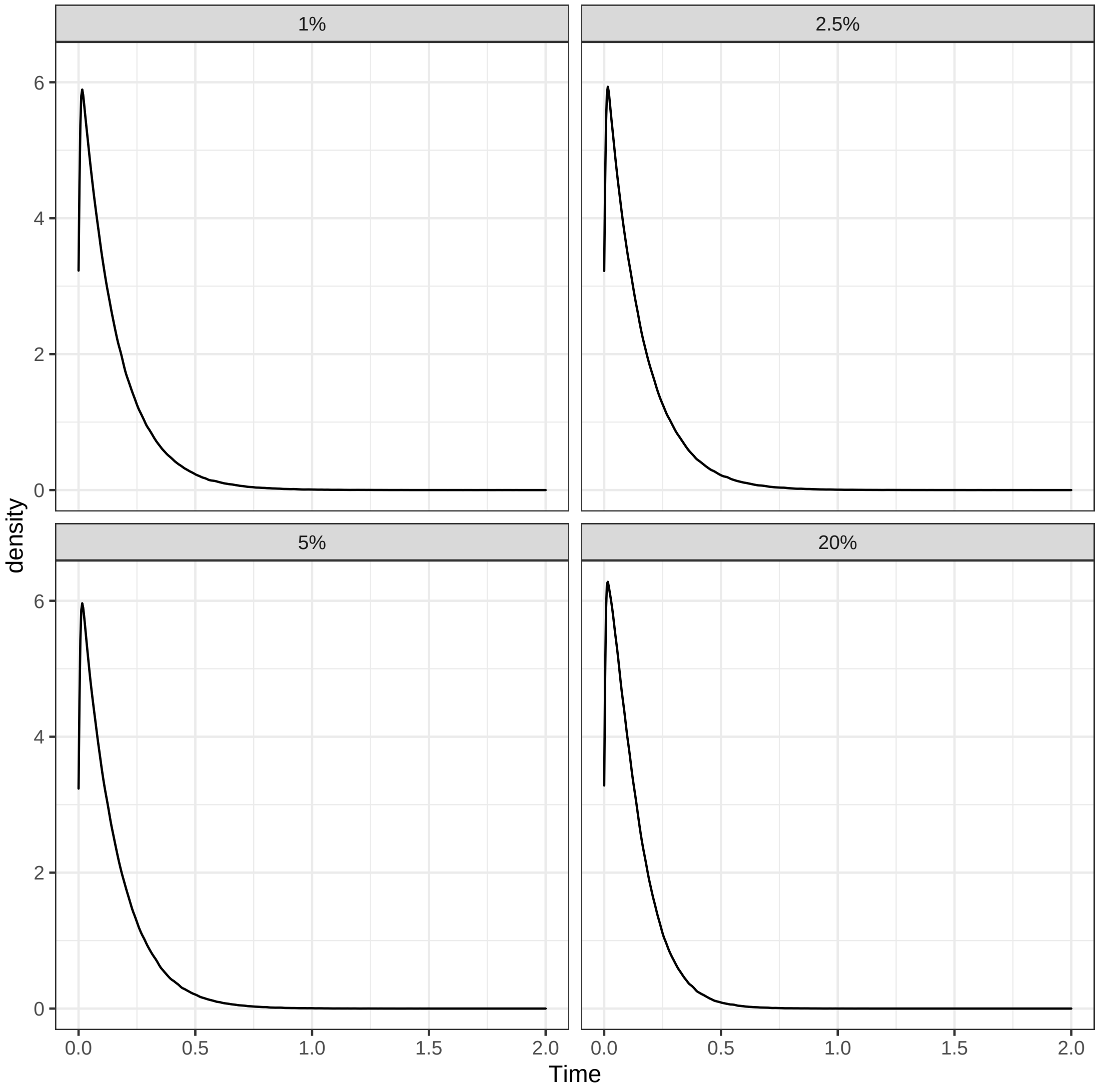

Supplementary Figure 3: **Observed event times in simulations for varying prevalences.** For all 1 million simulated individuals, a censoring, c, and onset $\tilde{t}$, is simulated. For a sub-sample of $20,000$, we illustrate the observed event time for different prevalences. The observed event time is derived as $t = \min(c, \tilde{t})$.

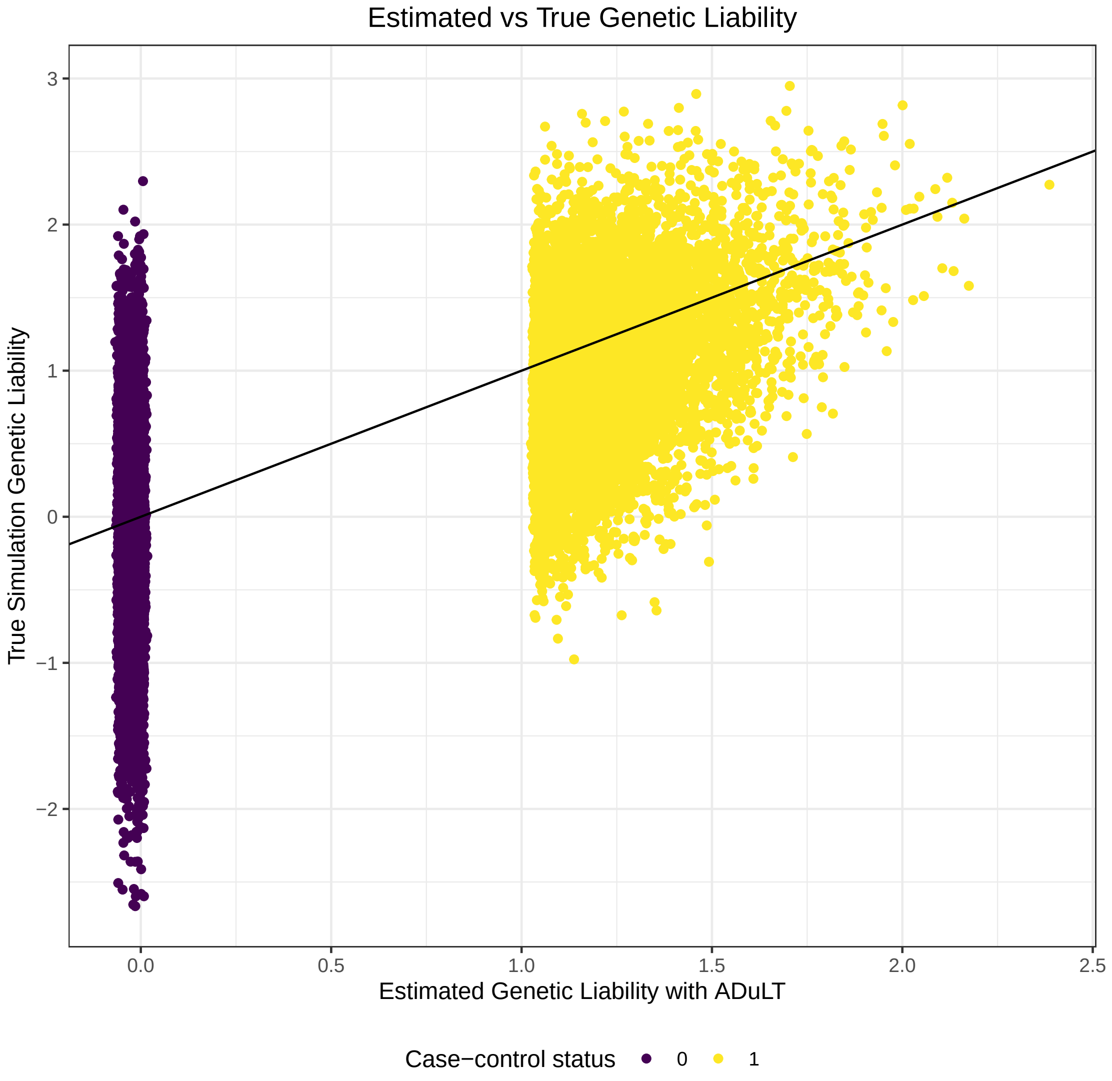

Supplementary Figure 4: **Estimated genetic liability from ADuLT.** For all 1 million simulated individuals, a censoring, c, and onset $\tilde{t}$, is simulated. From these, an observed event time is derived as $t = \min(c, \tilde{t})$. The observed event times are illustrated for different prevalences.

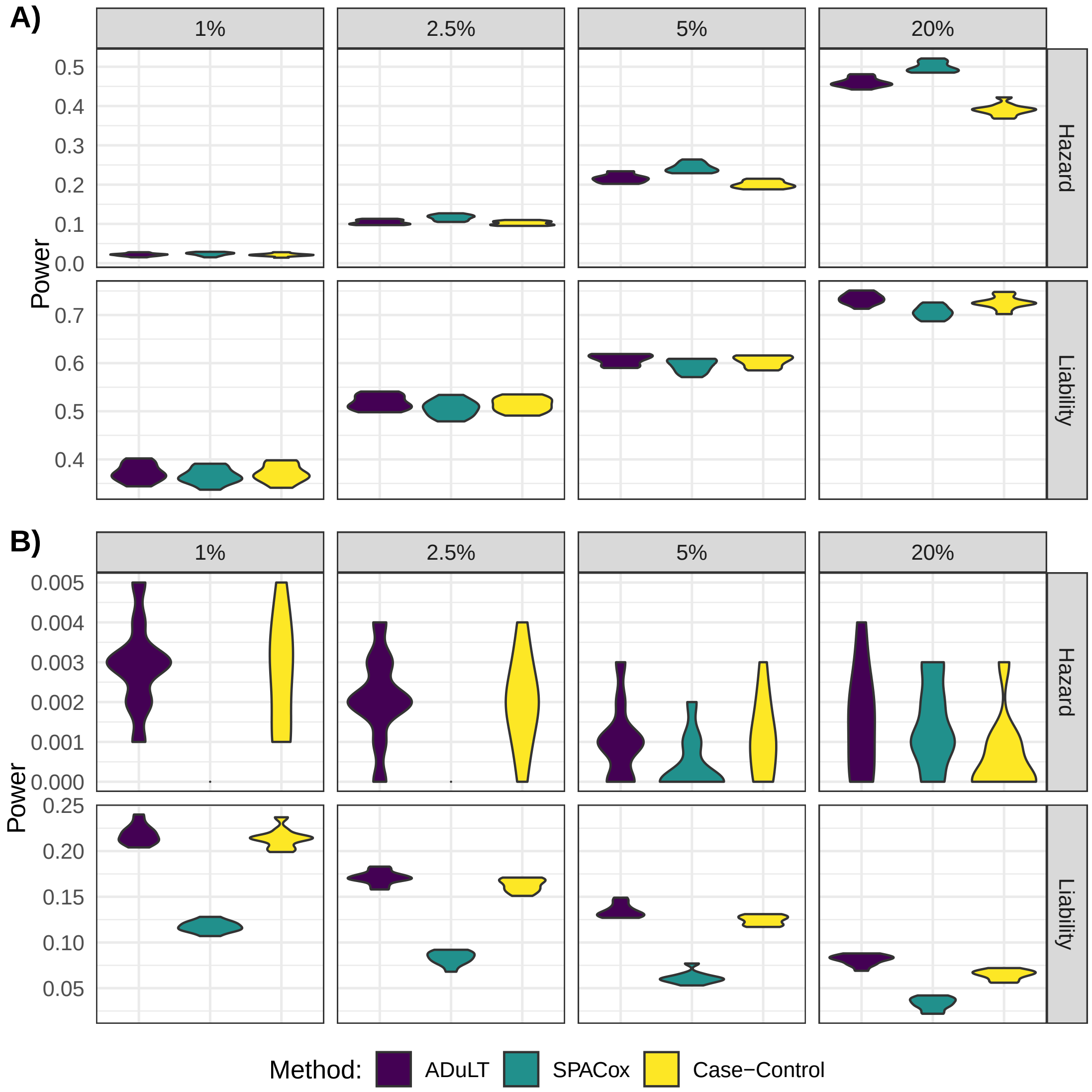

Supplementary Figure 5: **Power simulation results with 1000 causal SNPs under both generative models and varying prevalences.** The power, i.e. the fraction of causal SNPs detected for each of the three methods, is shown for several prevalences, varying from 1% to 20%. The generative model for `Hazard` is the proportional hazards model, and for `Liability` it is the liability threshold model. The simulation results are based on 10 replications. **A)** The power of ADuLT, SPACox and case-control GWAS **without downsampling**. **B)** The power for the same three methods but **with downsampling**. When downsampling, the number of individuals is set to 20k, with 10k cases and 10k controls. This is done to assess performance in biobanks where cases have been ascertained.

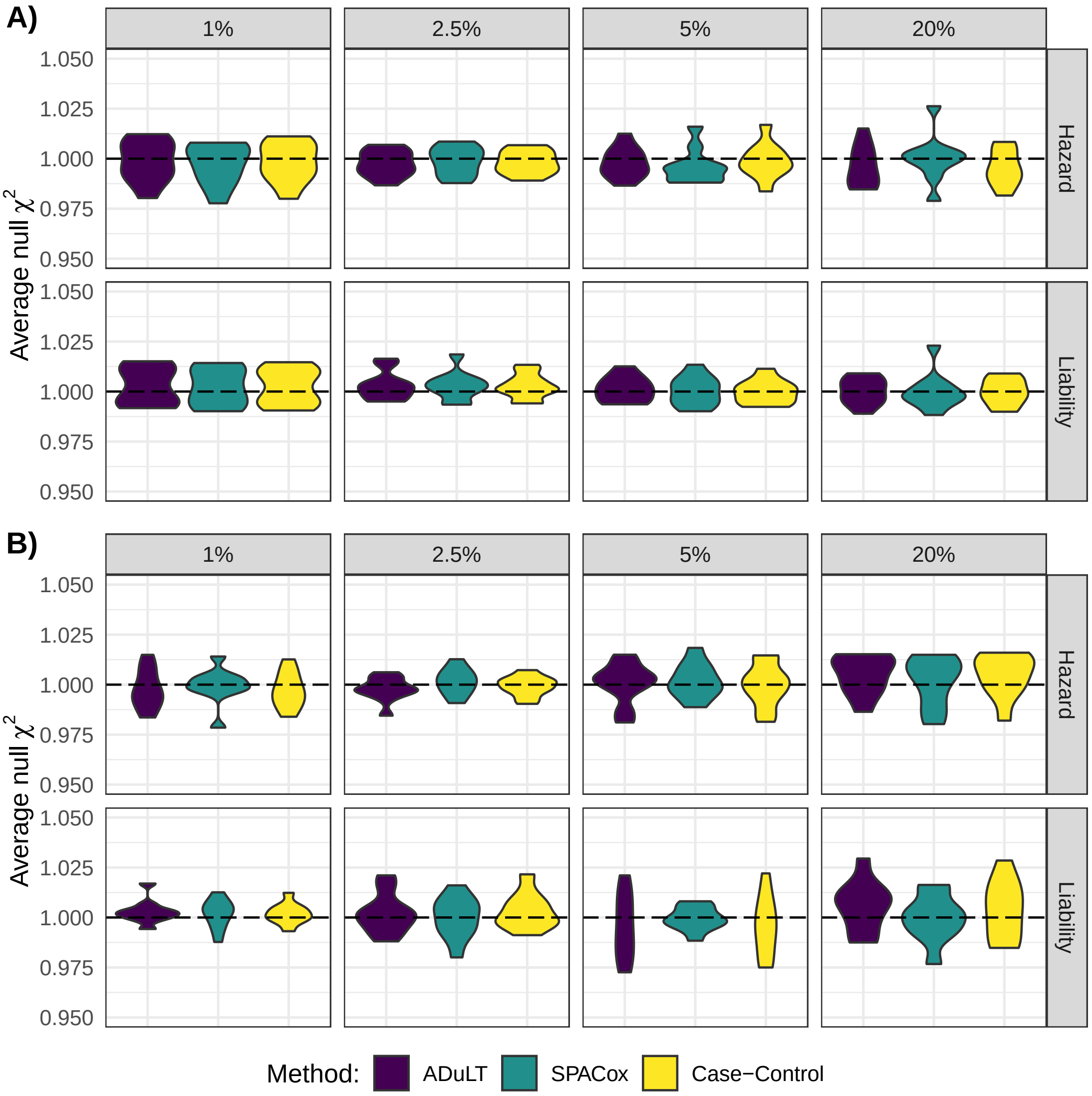

Supplementary Figure 6: **Average null SNPs χ^2-statistic simulation results with 250 causal SNPs under both generative models and varying prevalences.** The average null χ^2-statistic is shown for several prevalences, varying from 1% to 20%. The generative model for `Hazard` is the proportional hazards model, and for `Liability` it is the liability threshold model. **A)** The average null statistics for ADuLT, SPACox and case-control GWAS **without downsampling**. **B)** The average null statistics for the same three methods, but **with downsampling**. When downsampling, the number of individuals is set to 20k, with 10k cases and 10k controls. This is done to assess performance in biobanks where cases have been ascertained.

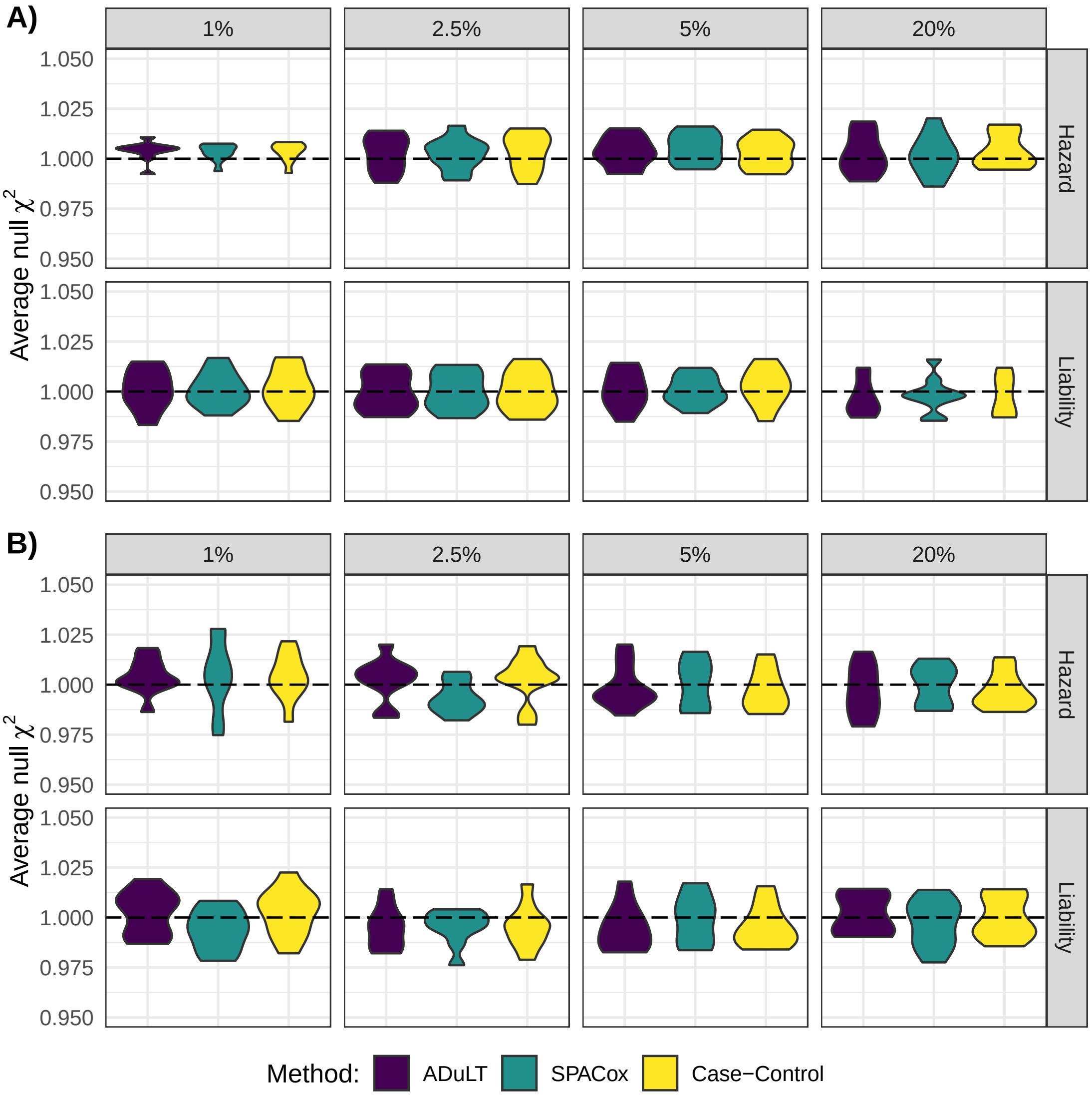

Supplementary Figure 7: **Average null SNPs χ^2-statistic simulation results with 1000 causal SNPs under both generative models and varying prevalences.** The average null χ^2-statistic is shown for several prevalences varying from 1% to 20%. The generative model for `Hazard` is the proportional hazards model, and for `Liability` it is the liability threshold model. **A)** The average null statistics for ADuLT, SPACox and case-control GWAS **without downsampling**. **B)** The average null statistics for the same three methods, but **with downsampling**. When downsampling, the number of individuals is set to 20k, with 10k cases and 10k controls. This is done to assess performance in biobanks where cases have been ascertained.

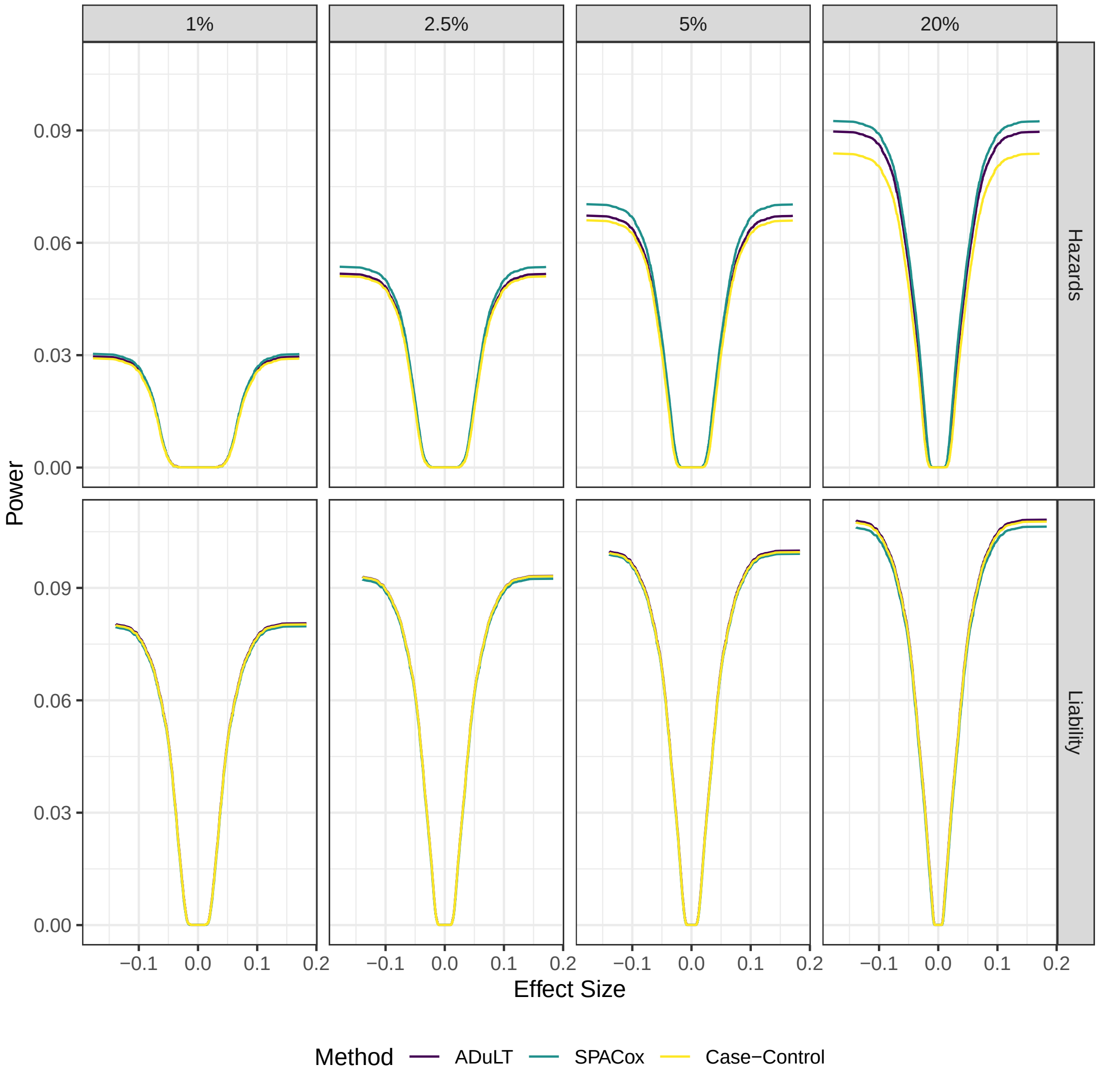

Supplementary Figure 8: **Power simulation results as a function of effect size with 250 causal SNPs under both generative models and varying prevalences.** The power, i.e. the fraction of causal SNPs detected for each of the three methods, is shown for several prevalences, varying from 1% to 20%. The generative model for `Hazard` is the proportional hazards model, and for `Liability` it is the liability threshold model. The simulation results are based on 10 replications.

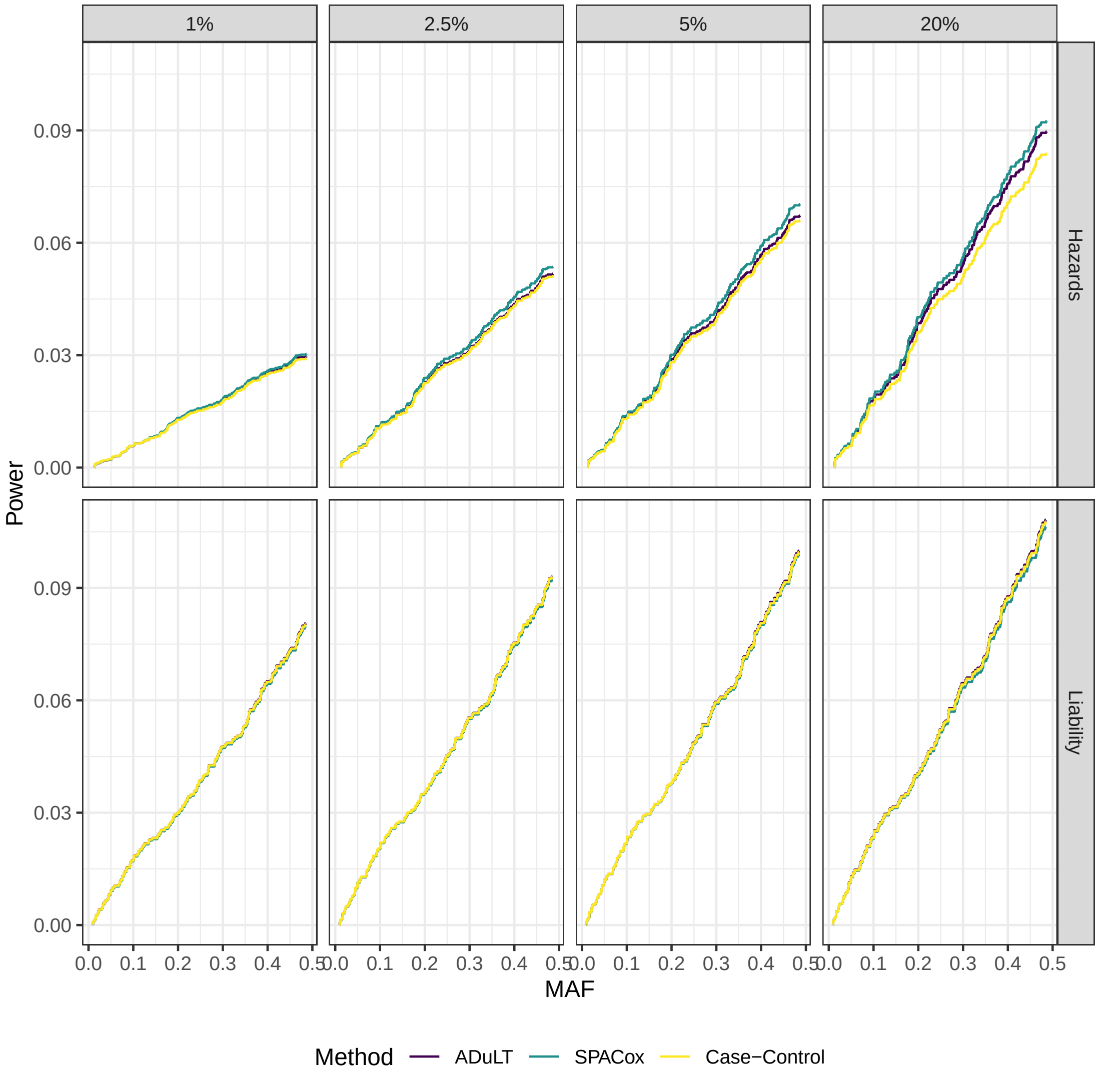

Supplementary Figure 9: **Power simulation results as a function of MAF with 250 causal SNPs under both generative models and varying prevalences.** The power, i.e. the fraction of causal SNPs detected for each of the three methods, is shown for several prevalences, varying from 1% to 20%. The generative model for `Hazard` is the proportional hazards model, and for `Liability` it is the liability threshold model. The simulation results are based on 10 replications.

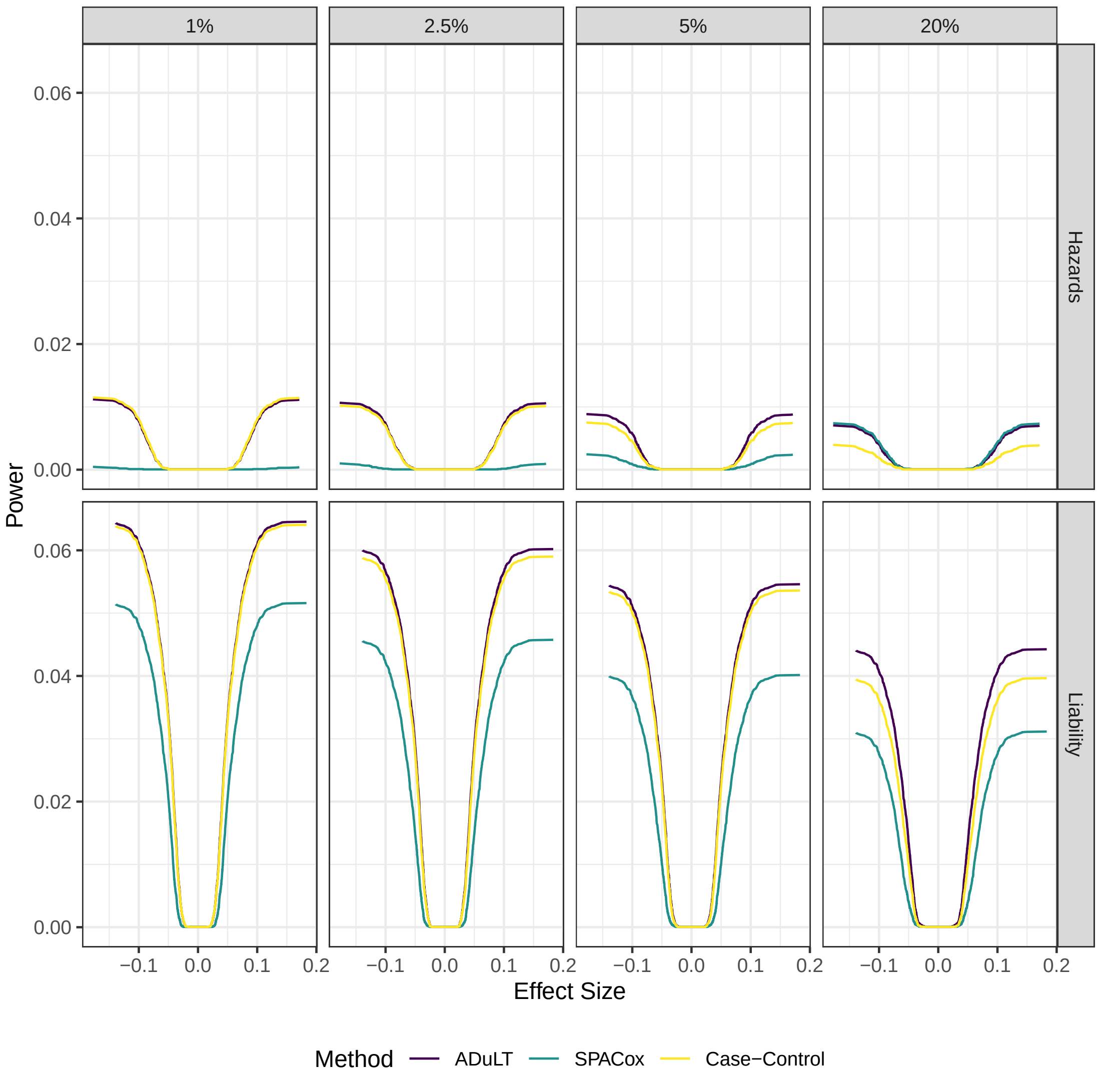

Supplementary Figure 10: **Power simulation results as a function of effect size with 250 causal SNPs under both generative models, varying prevalences, and with case ascertainment.** The power, i.e. the fraction of causal SNPs detected for each of the three methods, is shown for several prevalences, varying from 1% to 20%. The generative model for `Hazard` is the proportional hazards model, and for `Liability` it is the liability threshold model. The simulation results are based on 10 replications. When case ascertainment is present, the number of individuals is set to 20k, with 10k cases and 10k controls. This is done to assess performance in biobanks where cases have been ascertained.

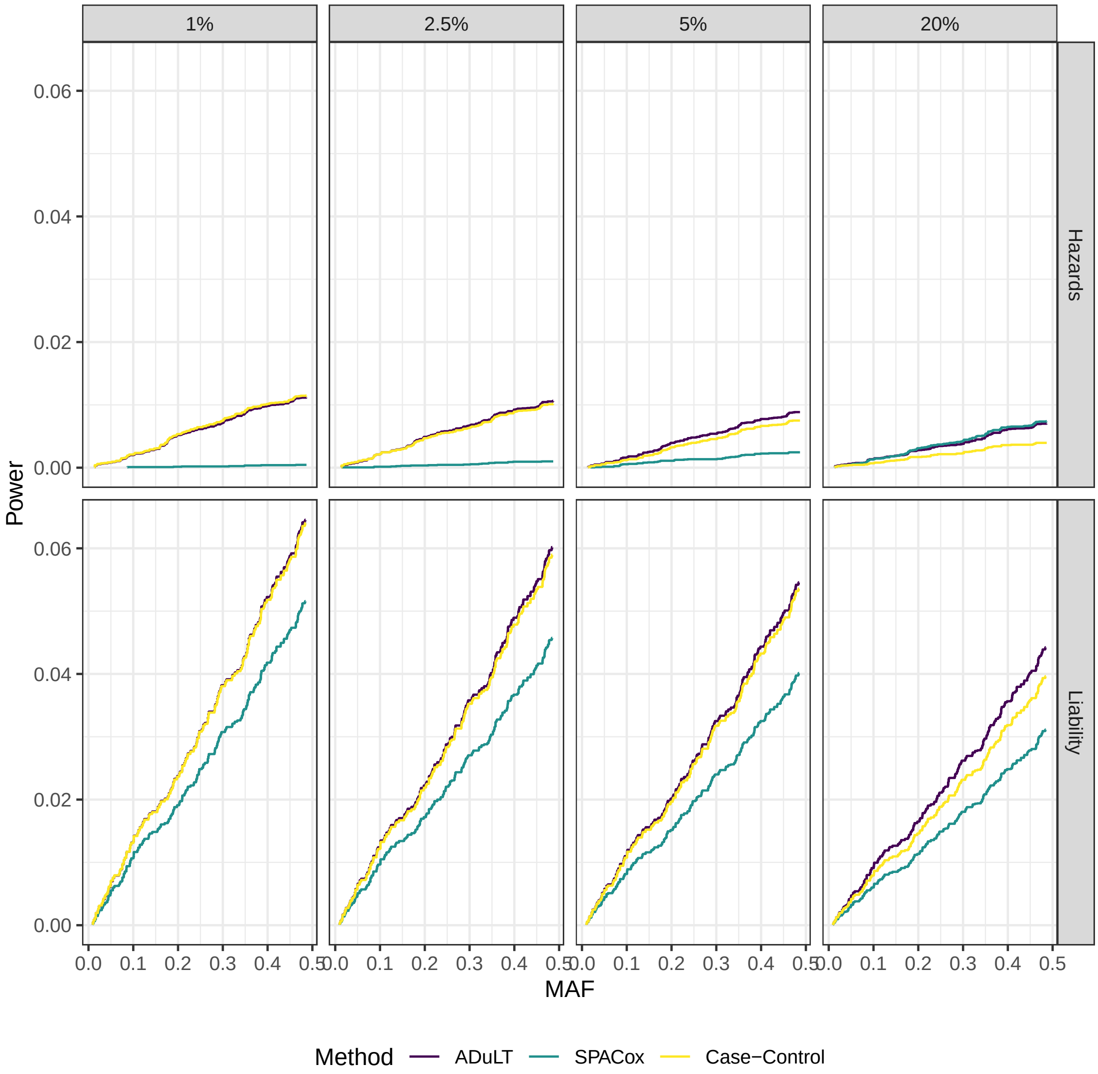

Supplementary Figure 11: **Power simulation results as a function of MAF with 250 causal SNPs under both generative models, varying prevalences, and with case ascertainment.** The power, i.e. the fraction of causal SNPs detected for each of the three methods, is shown for several prevalences, varying from 1% to 20%. The generative model for `Hazard` is the proportional hazards model, and for `Liability` it is the liability threshold model. The simulation results are based on 10 replications. When case ascertainment is present, the number of individuals is set to 20k, with 10k cases and 10k controls. This is done to assess performance in biobanks where cases have been ascertained.

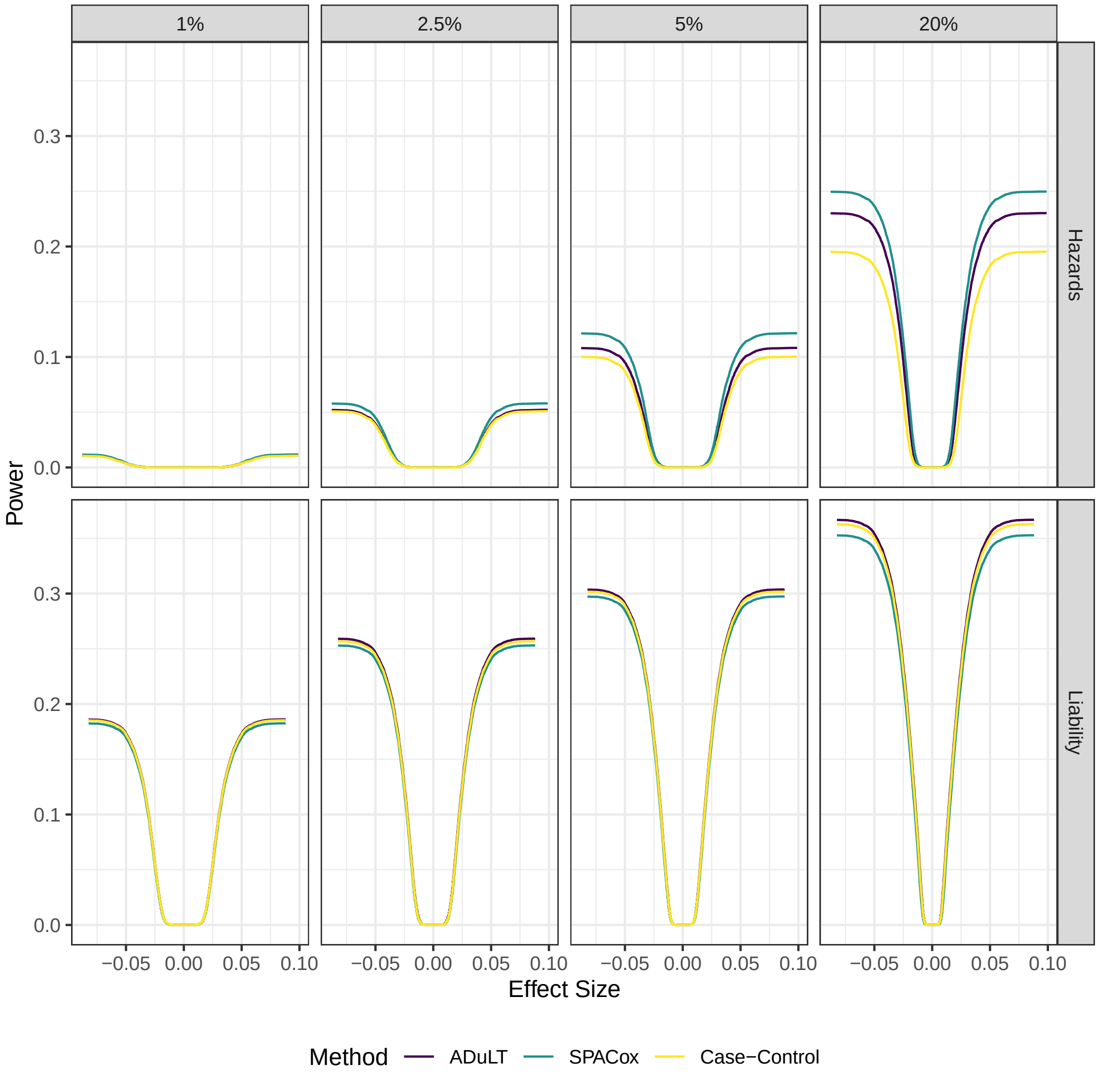

Supplementary Figure 12: **Power simulation results as a function of effect size with 1000 causal SNPs under both generative models and varying prevalences.** The power, i.e. the fraction of causal SNPs detected for each of the three methods, is shown for several prevalences, varying from 1% to 20%. The generative model for `Hazard` is the proportional hazards model, and for `Liability` it is the liability threshold model. The simulation results are based on 10 replications.

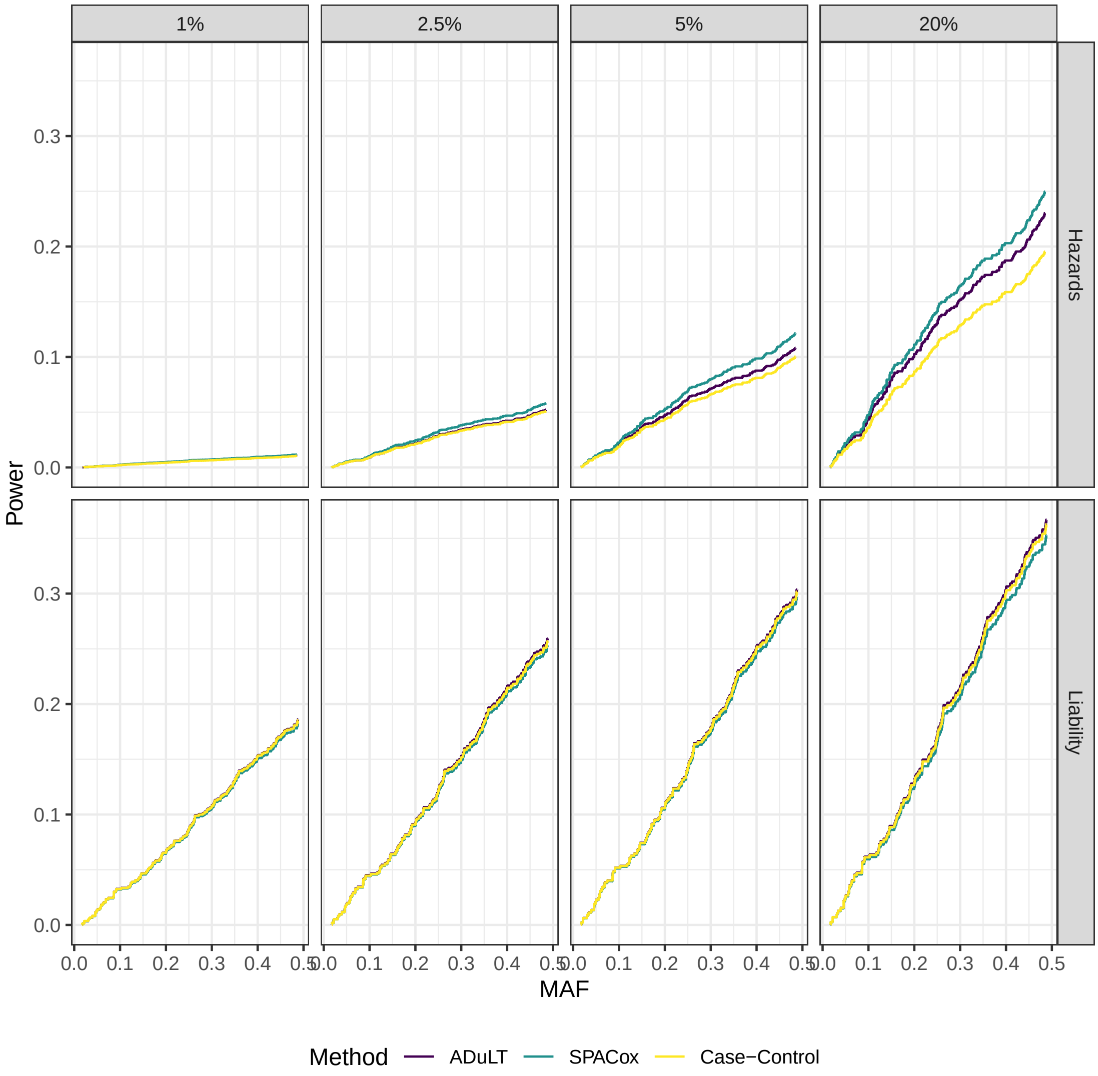

Supplementary Figure 13: **Power simulation results as a function of MAF with** 1000 **causal SNPs under both generative models and varying prevalences.** The power, i.e. the fraction of causal SNPs detected for each of the three methods, is shown for several prevalences, varying from 1% to 20%. The generative model for `Hazard` is the proportional hazards model, and for `Liability` it is the liability threshold model. The simulation results are based on 10 replications.

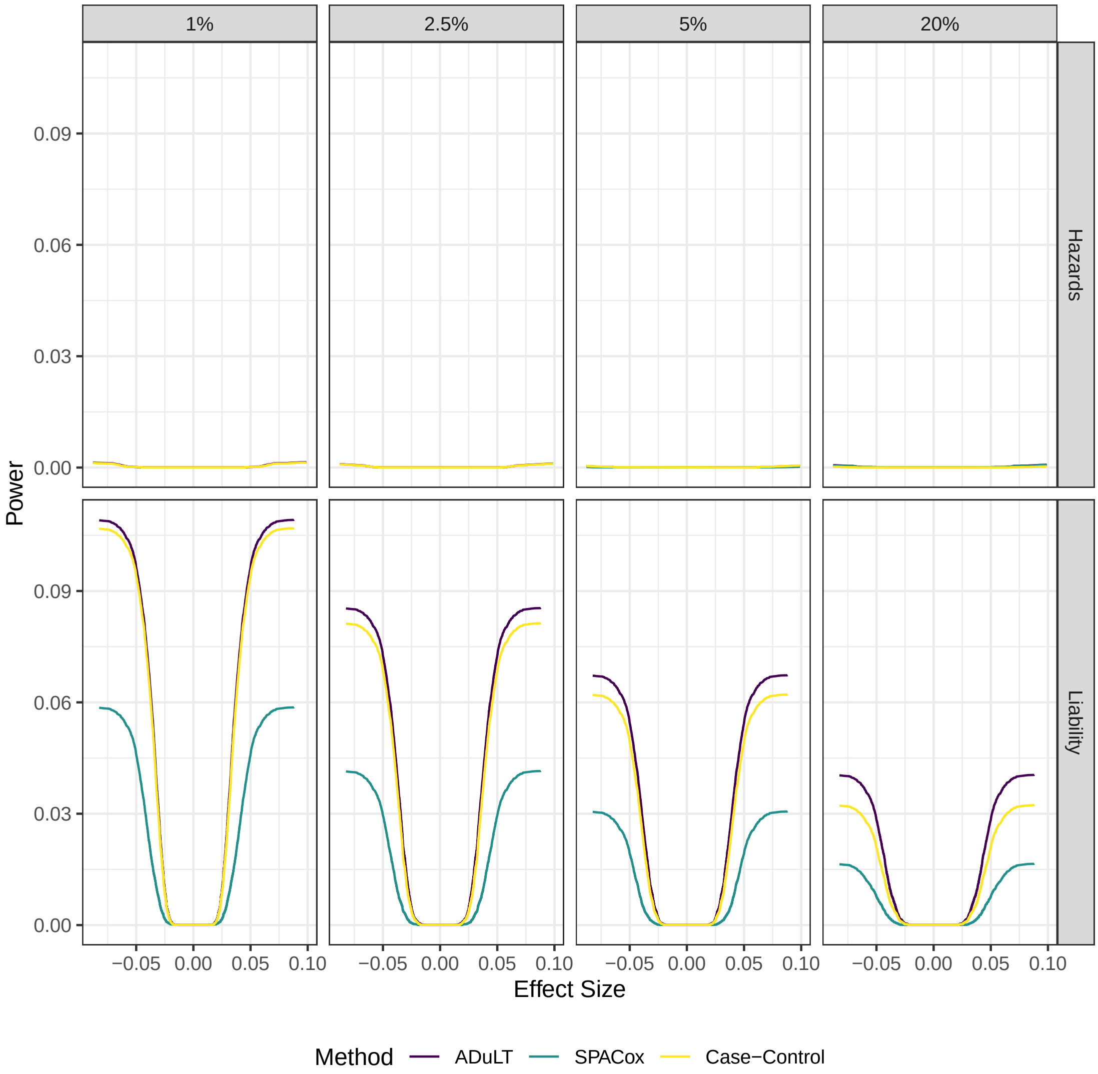

Supplementary Figure 14: **Power simulation results as a function of effect size with 1000 causal SNPs under both generative models, varying prevalences, and with case ascertainment.** The power, i.e. the fraction of causal SNPs detected for each of the three methods, is shown for several prevalences, varying from 1% to 20%. The generative model for `Hazard` is the proportional hazards model, and for `Liability` it is the liability threshold model. The simulation results are based on 10 replications. When case ascertainment is present, the number of individuals is set to 20k, with 10k cases and 10k controls. This is done to assess performance in biobanks where cases have been ascertained.

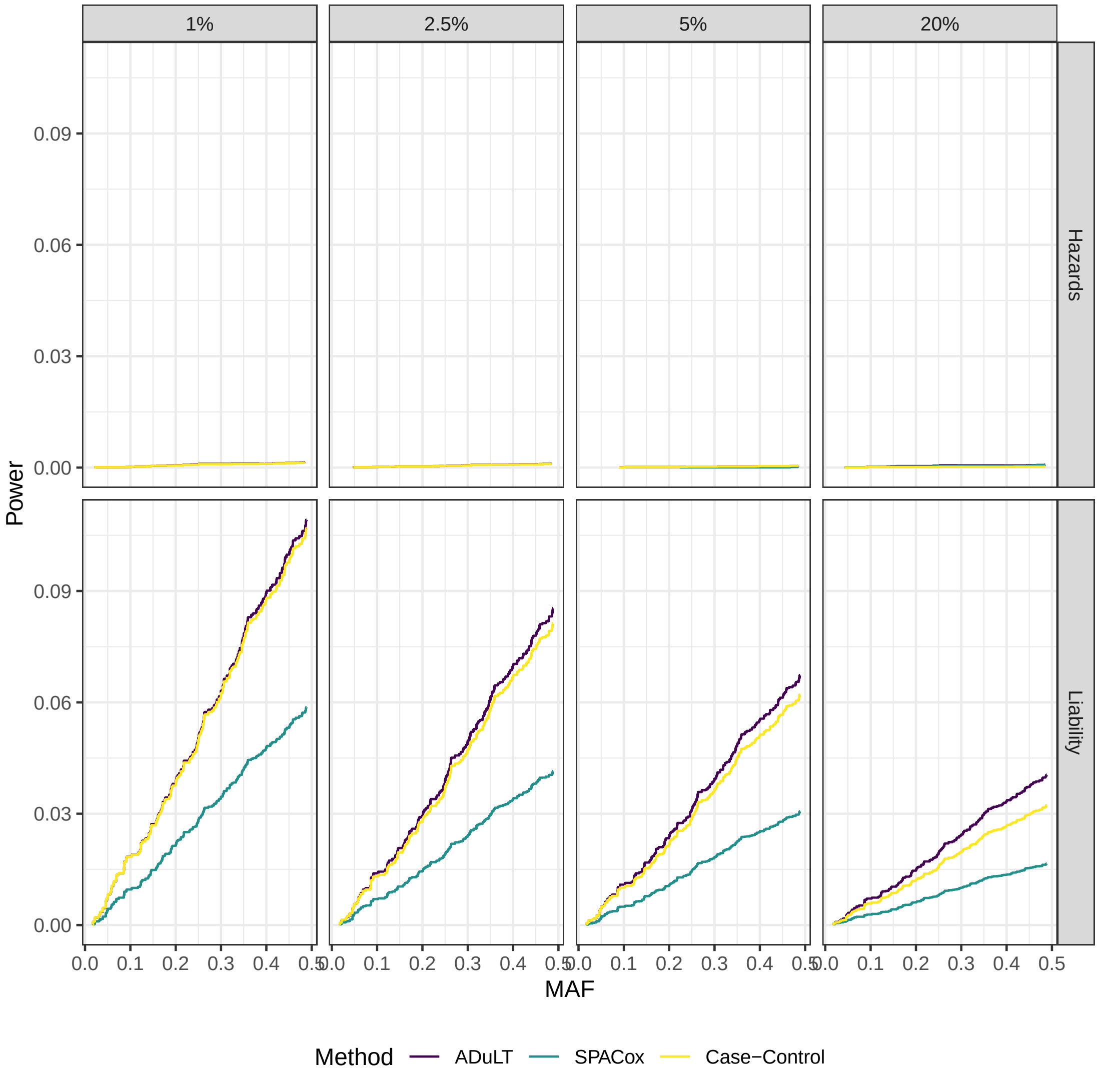

Supplementary Figure 15: **Power simulation results as a function of MAF with** 1000 **causal SNPs under both generative models, varying prevalences, and with case ascertainment.** The power, i.e. the fraction of causal SNPs detected for each of the three methods, is shown for several prevalences, varying from 1% to 20%. The generative model for `Hazard` is the proportional hazards model, and for `Liability` it is the liability threshold model. The simulation results are based on 10 replications. When case ascertainment is present, the number of individuals is set to 20k, with 10k cases and 10k controls. This is done to assess performance in biobanks where cases have been ascertained.

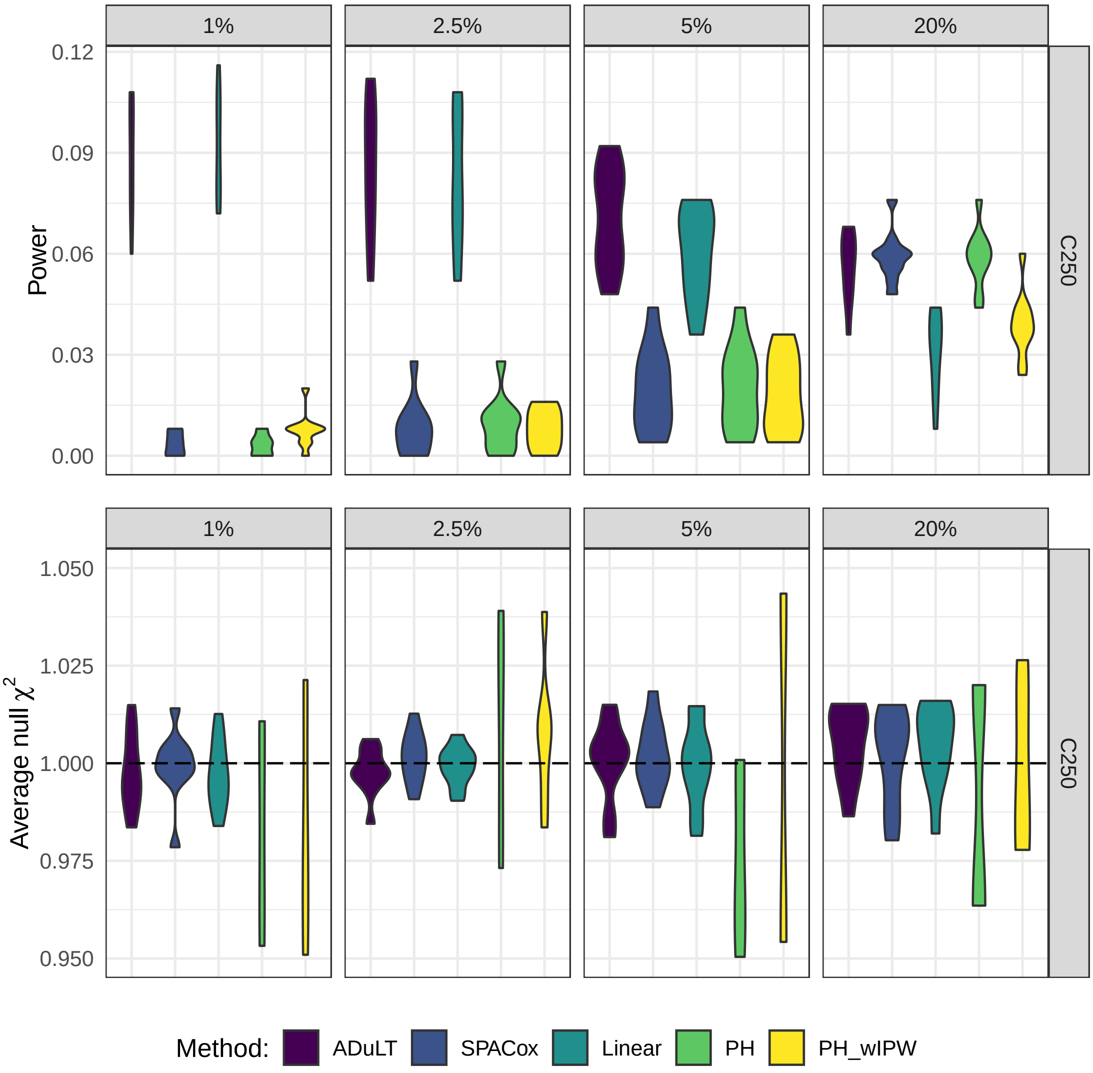

Supplementary Figure 16: **Simulation results with inverse probability weighing for 250 causal SNPs and downsampling.** Under the proportional hazards model, the power and average null statistic are shown for several prevalences varying from 1% to 20%. Here **PH** refers to the proportional hazards implementation from the `survival` package in `R`[1]. The implementation supports weighs, and **PH_wIPW** is the proportional hazards models with inverse probability weighs. When case ascertainment is present, the number of individuals is set to 20k, with 10k cases and 10k controls. This is done to assess performance in biobanks where cases have been ascertained. 10 replications were performed for each parameter setup.

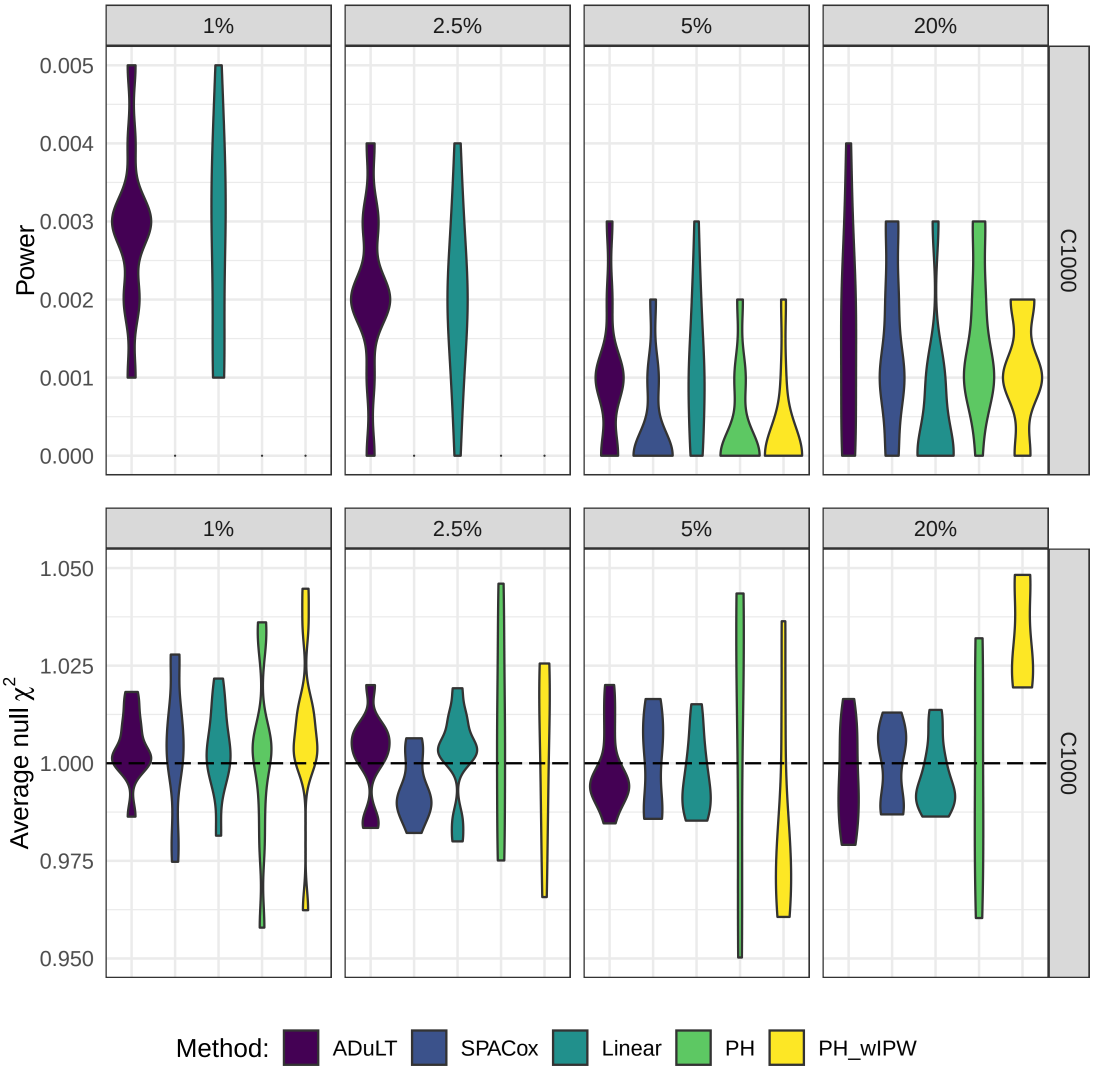

Supplementary Figure 17: **Simulation results with inverse probability weighing for 1000 causal SNPs and downsampling.** Under the proportional hazards model, the power and average null statistic are shown for several prevalences varying from 1% to 20%. Here **PH** refers to the proportional hazards implementation from the `survival` package in `R`[1]. The implementation supports weighs, and **PH_wIPW** is the proportional hazards models with inverse probability weighs. When case ascertainment is present, the number of individuals is set to 20k, with 10k cases and 10k controls. This is done to assess performance in biobanks where cases have been ascertained. 10 replications were performed for each parameter setup.

iPSYCH Results

ADHD

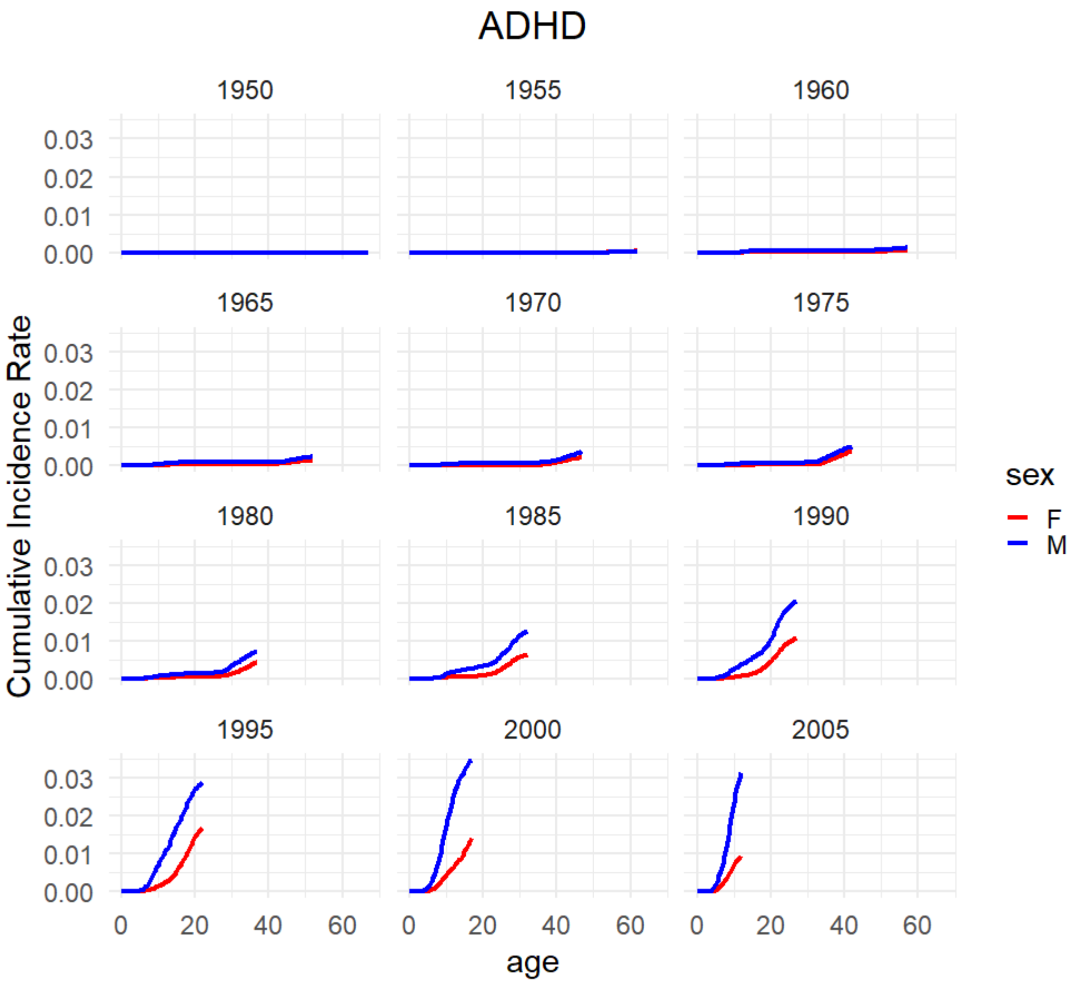

Supplementary Figure 18: **Cumulative incidence rates for ADHD.** Cumulative incidence rates for ADHD in the Danish registers. The cumulative incidence proportions are stratified by birth year and sex.

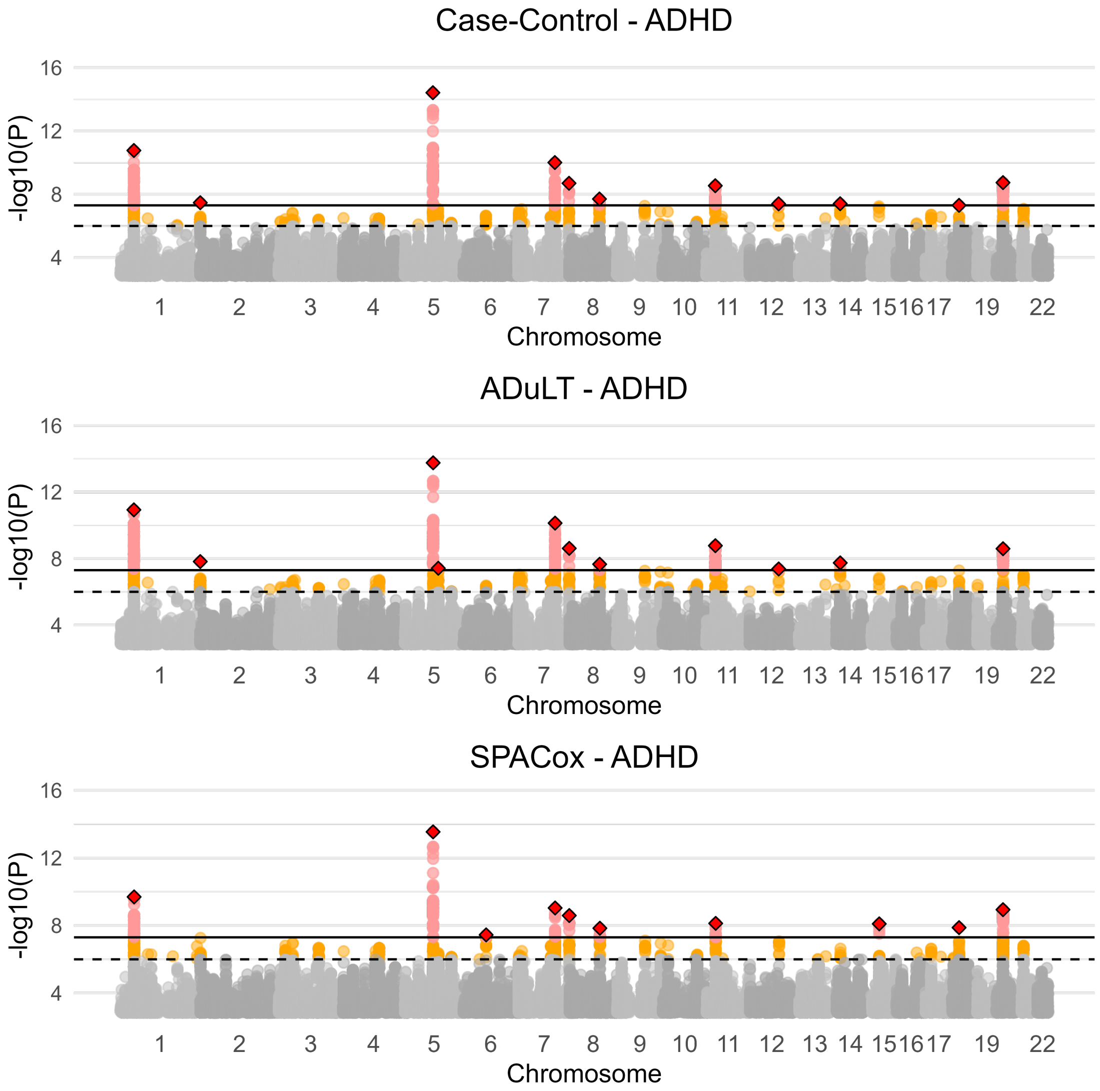

Supplementary Figure 19: **Manhattan plots for SPACox as well as for a GWAS with ADuLT and case-control status as the outcome for ADHD with age as a covariate for all phenotypes.** Manhattan plots for ADHD based on the ADuLT GWAS, case-control GWAS and SPACox. The orange dots indicate suggestive SNPs with a p-value threshold of 5×10^{-6}. The red dots correspond to Bonferroni-adjusted genome-wide significant SNPs with a p-value threshold of 5×10^{-8}. The diamonds correspond to the lowest p-value LD clumped SNP in a 500k base pair window with an $r^2 = 0.1$ threshold. All tests performed are two-sided.

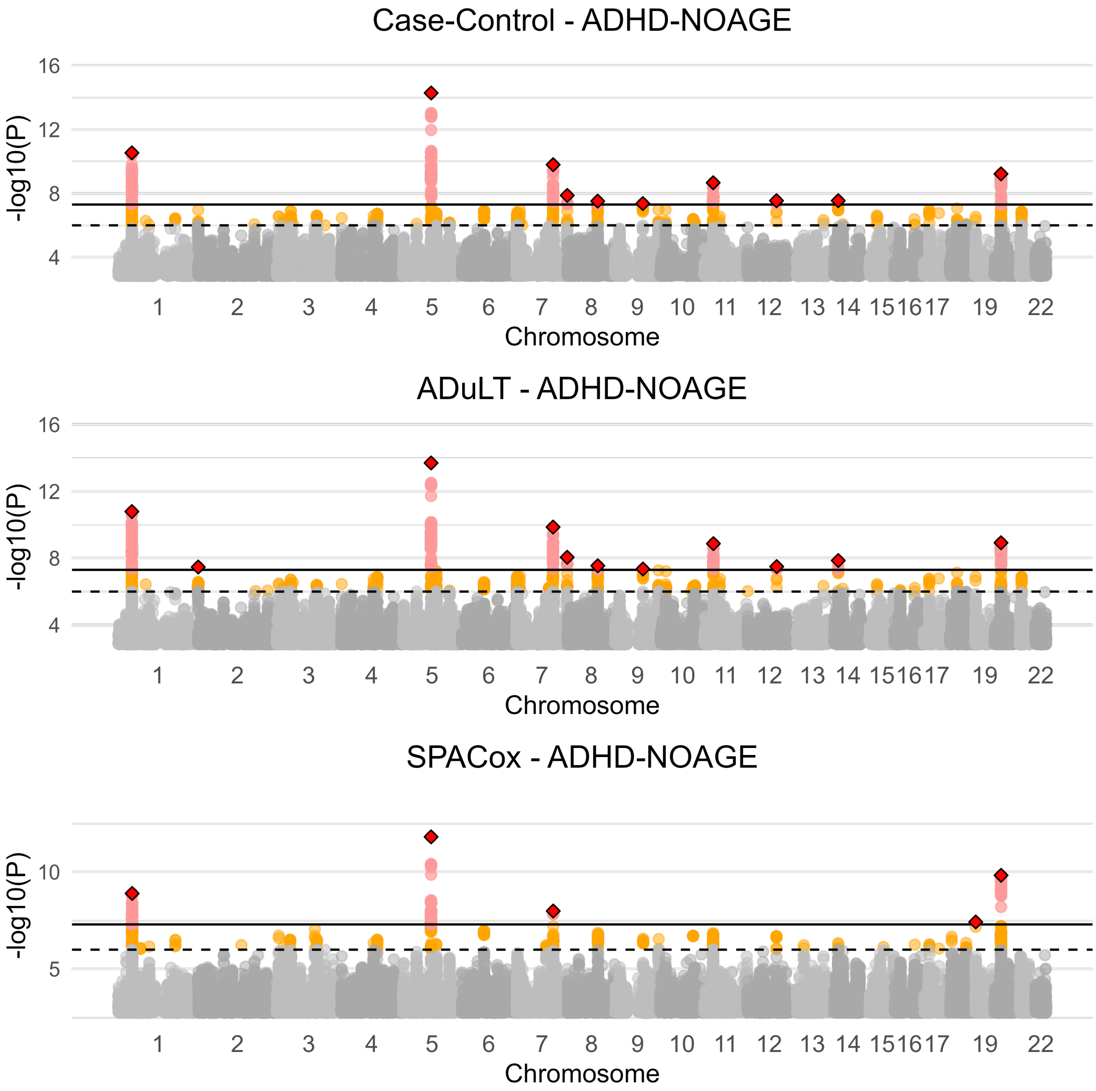

Supplementary Figure 20: **Manhattan plots for ADuLT, case-control status, and SPACox of ADHD without age as a covariate for all phenotypes.** Manhattan plots for ADHD using the three methods. The orange dots indicate suggestive SNPs with a p-value threshold of 5×10^{-6}. The red dots correspond to Bonferroni-adjusted genome-wide significant SNPs with a p-value threshold of 5×10^{-8}. The diamonds correspond to the lowest p-value LD clumped SNP in a 500k base pair window with an $r^2 = 0.1$ threshold. All tests performed are two-sided.

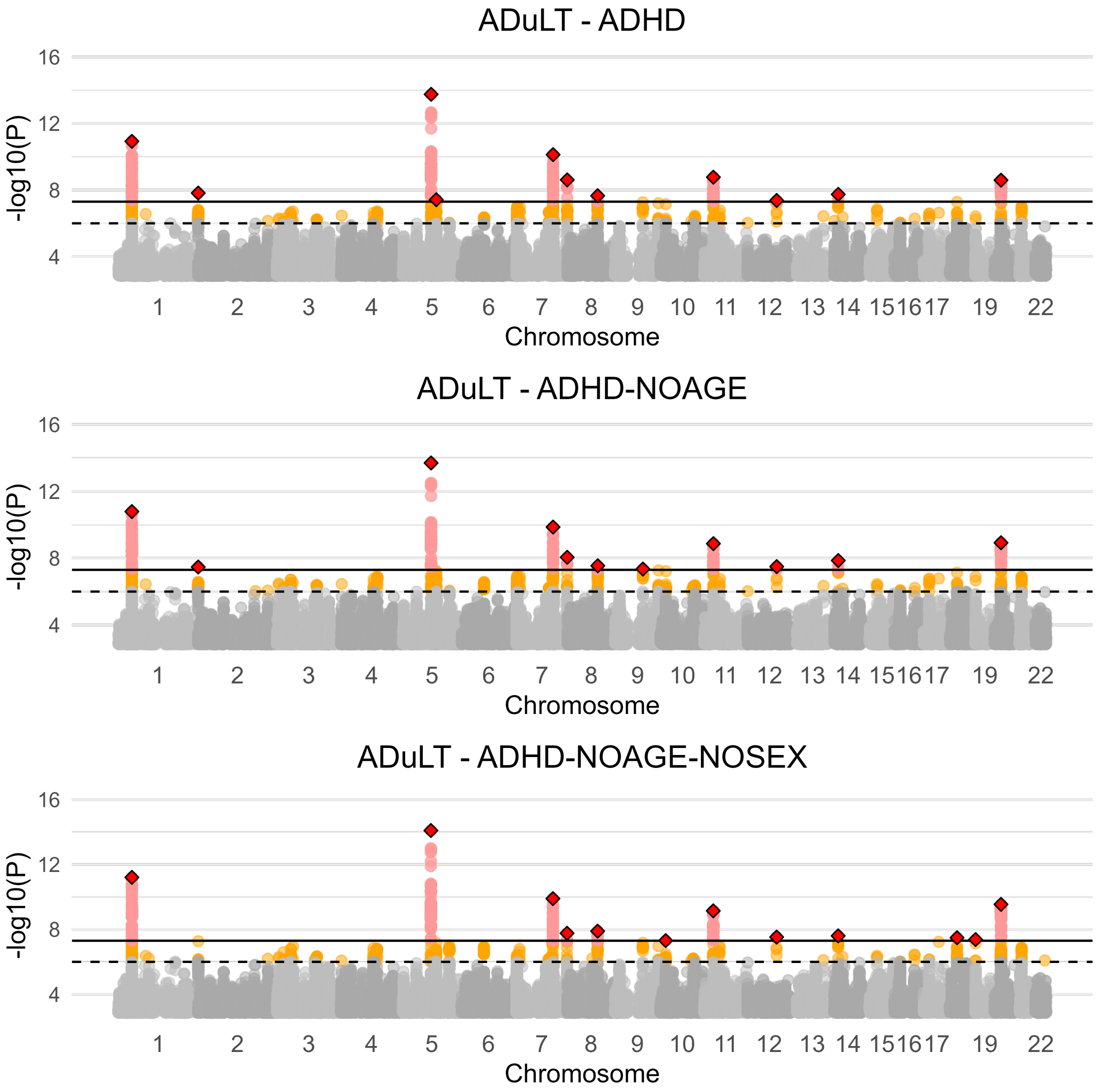

Supplementary Figure 21: **Manhattan plots for ADuLT with different included covariates.** Manhattan plots for ADHD using the ADuLT phenotype. The Manhattan plots are with age and sex as covariates, without age, and without both sex and age. The orange dots indicate suggestive SNPs with a p-value threshold of 5×10^{-6}. The red dots correspond to Bonferroni-adjusted genome-wide significant SNPs with a p-value threshold of 5×10^{-8}. The diamonds correspond to the lowest p-value LD clumped SNP in a 500k base pair window with an $r^2 = 0.1$ threshold. All tests performed are two-sided.

Autism

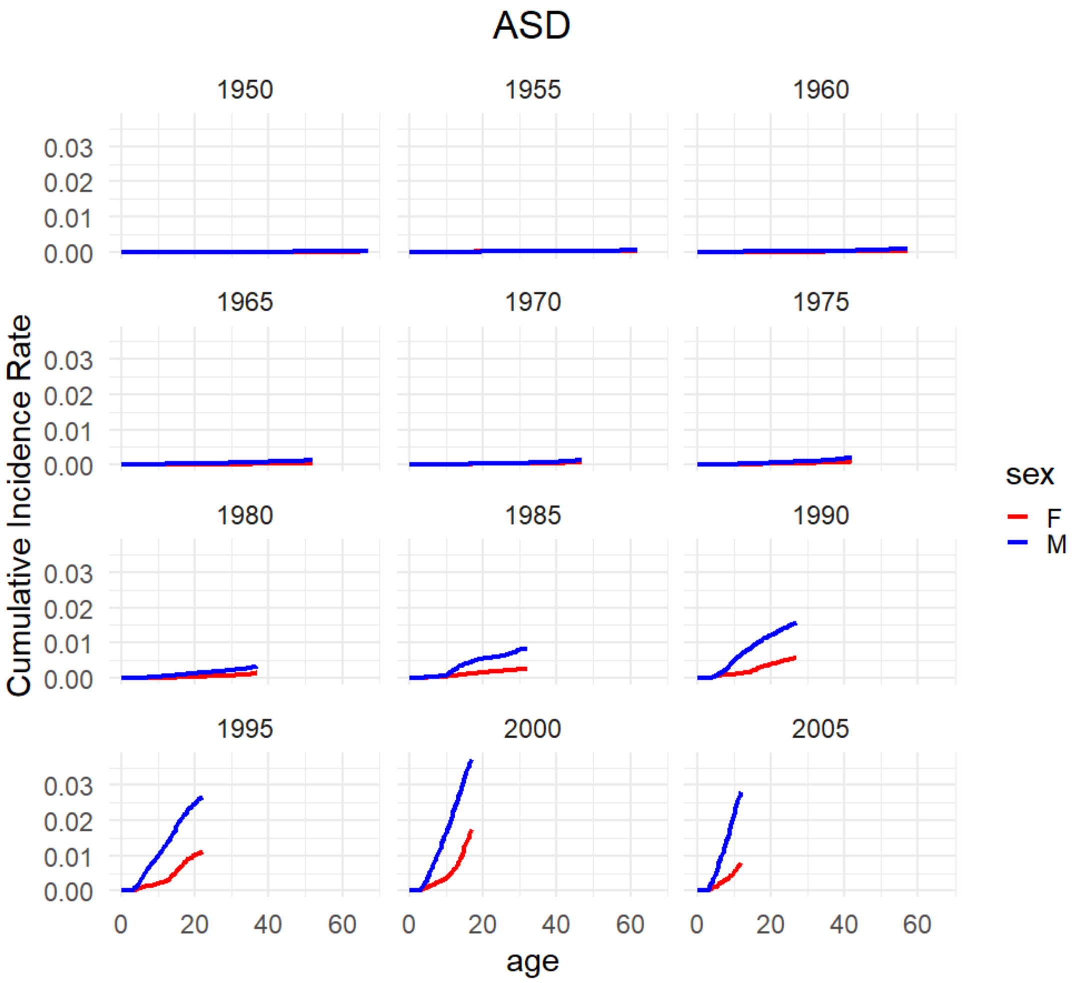

Supplementary Figure 22: **Cumulative incidence rates for Autism.** Cumulative incidence rates for autism in the Danish registers. The cumulative incidence proportions are stratified by birth year and sex.

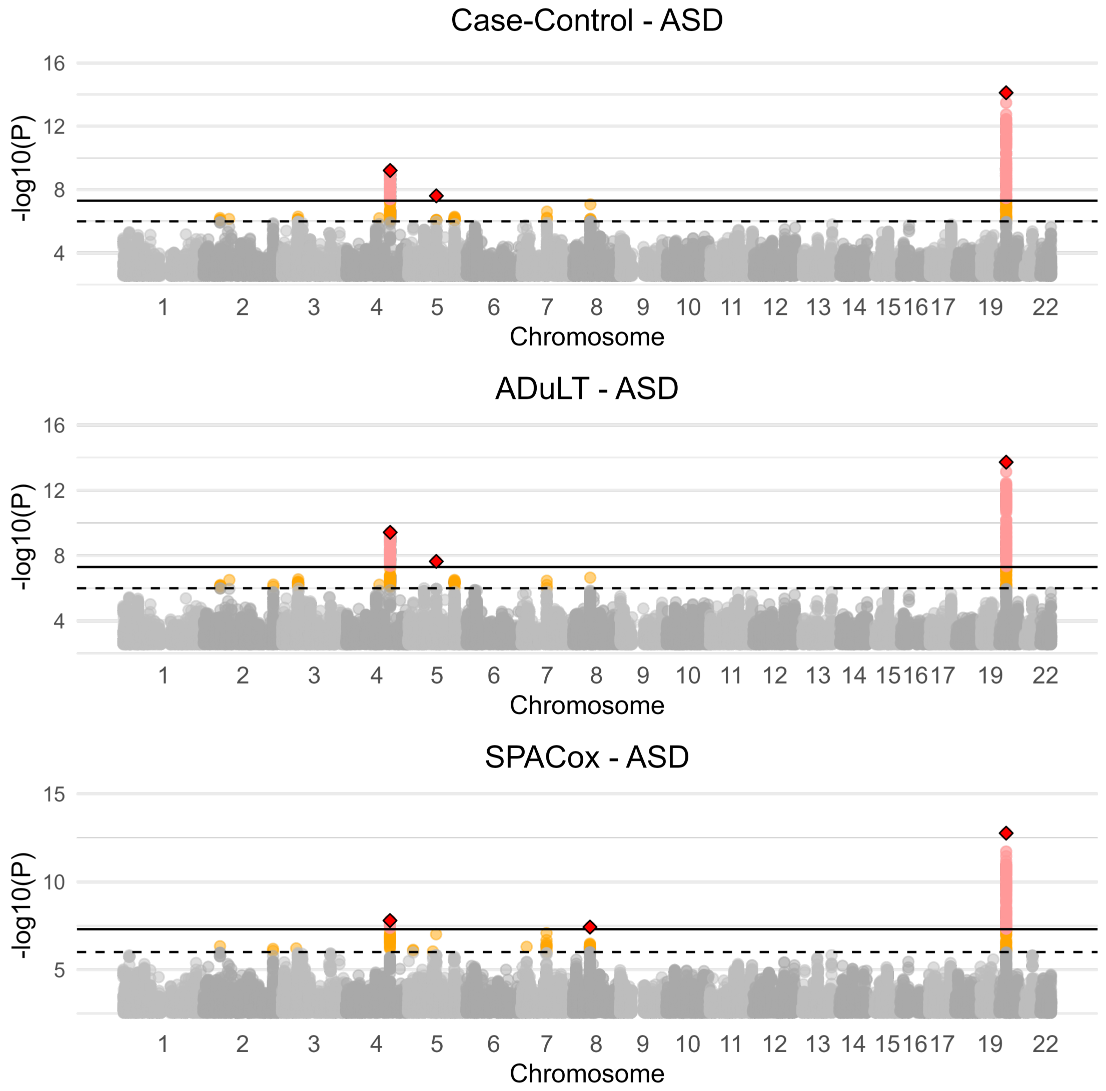

Supplementary Figure 23: **Manhattan plots for ADuLT, case-control status, and SPACox of autism with age as a covariate for all phenotypes.** Manhattan plots for autism using the three methods. The orange dots indicate suggestive SNPs with a p-value threshold of 5×10^{-6}. The red dots correspond to Bonferroni-adjusted genome-wide significant SNPs with a p-value threshold of 5×10^{-8}. The diamonds correspond to the lowest p-value LD clumped SNP in a 500k base pair window with an $r^2 = 0.1$ threshold. All tests performed are two-sided.

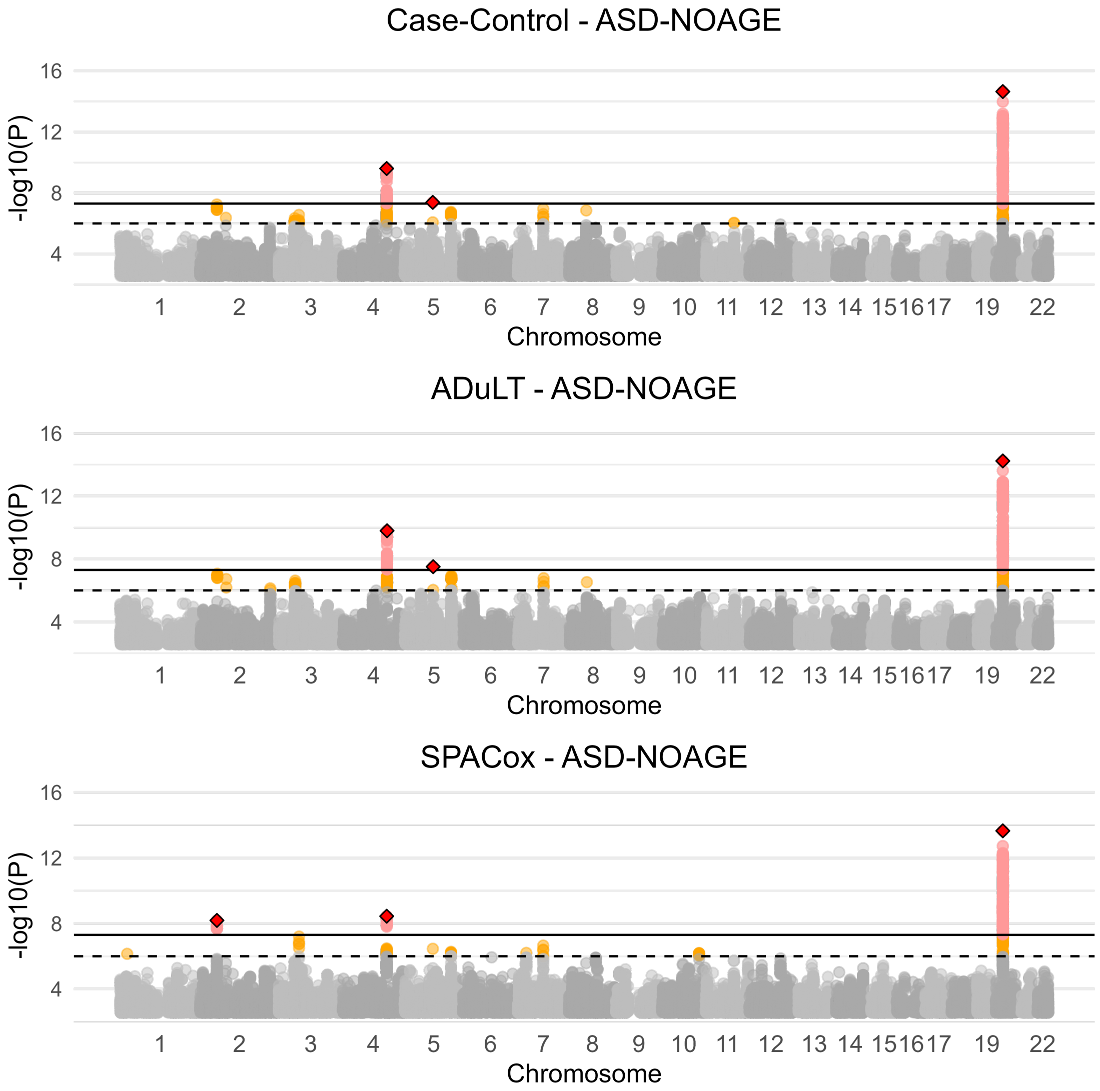

Supplementary Figure 24: **Manhattan plots for ADuLT, case-control status, and SPACox of autism without age as a covariate for all phenotypes.** Manhattan plots for autism using the three methods. The orange dots indicate suggestive SNPs with a p-value threshold of 5×10^{-6}. The red dots correspond to Bonferroni-adjusted genome-wide significant SNPs with a p-value threshold of 5×10^{-8}. The diamonds correspond to the lowest p-value LD clumped SNP in a 500k base pair window with an $r^2 = 0.1$ threshold. All tests performed are two-sided.

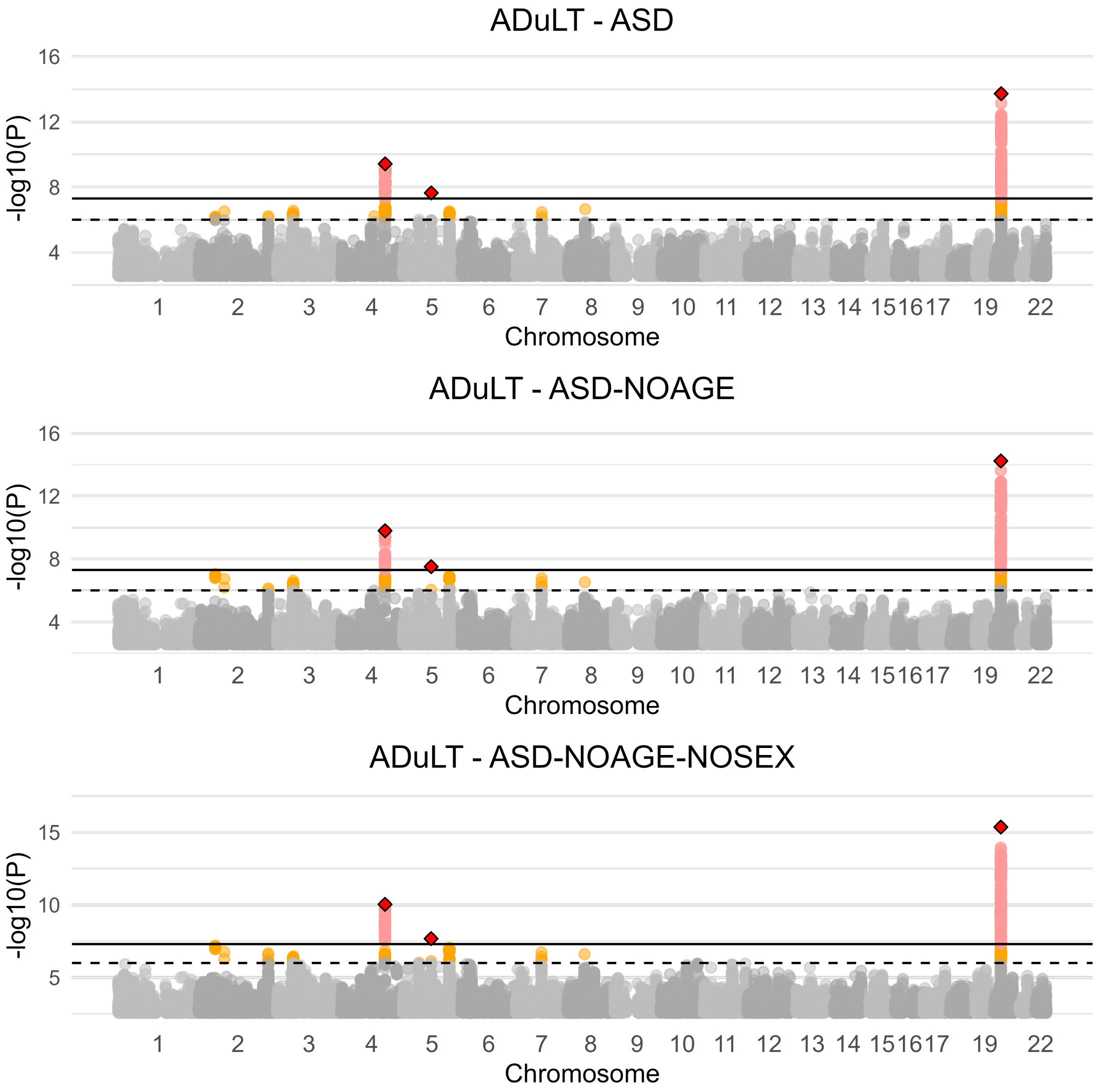

Supplementary Figure 25: **Manhattan plots for ADuLT with different included covariates.** Manhattan plots for autism using the ADuLT phenotype. The Manhattan plots are with age and sex as covariates, without age, and without both sex and age. The orange dots indicate suggestive SNPs with a p-value threshold of 5×10^{-6}. The red dots correspond to Bonferroni-adjusted genome-wide significant SNPs with a p-value threshold of 5×10^{-8}. The diamonds correspond to the lowest p-value LD clumped SNP in a 500k base pair window with an $r^2 = 0.1$ threshold. All tests performed are two-sided.

Depression

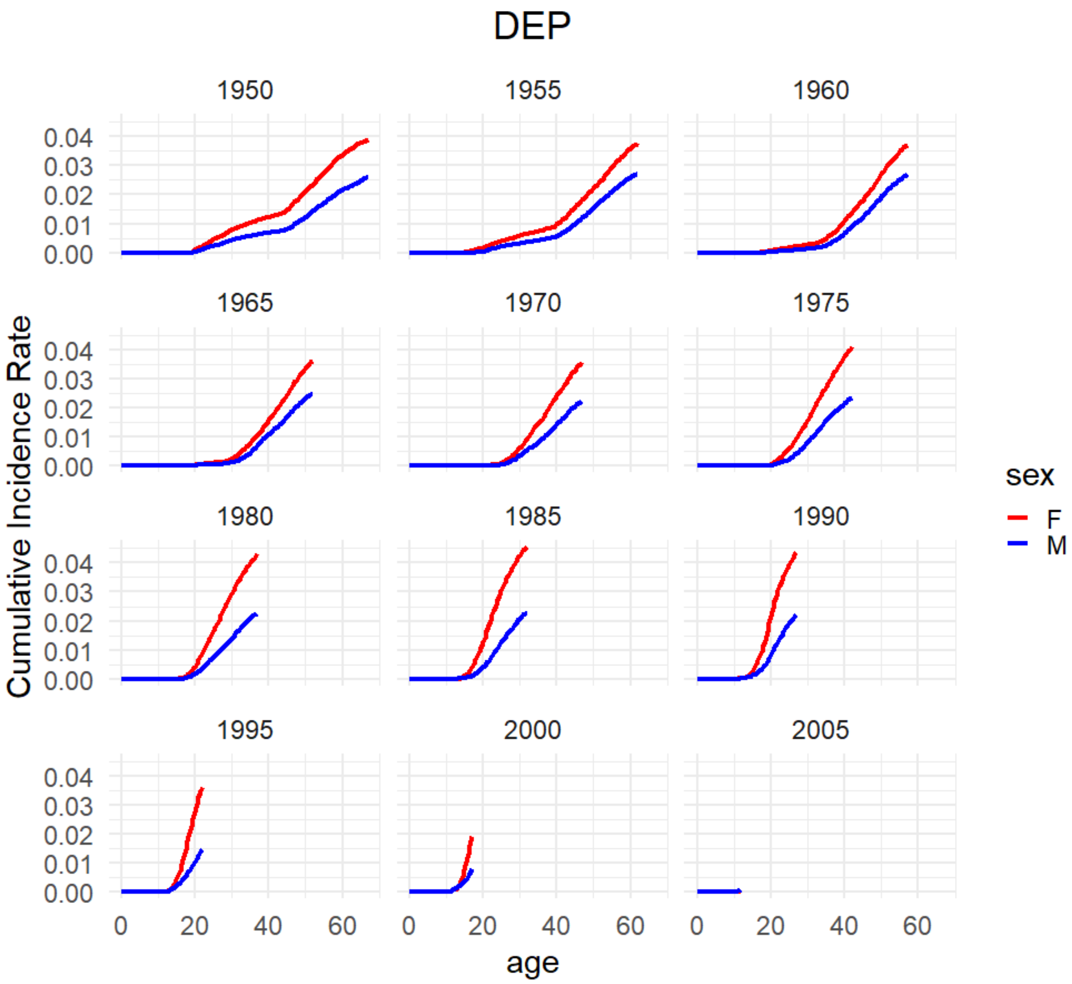

Supplementary Figure 26: **Cumulative incidence rates for Depression.** Cumulative incidence rates for depression in the Danish registers. The cumulative incidence proportions are stratifiedby birth year and sex.

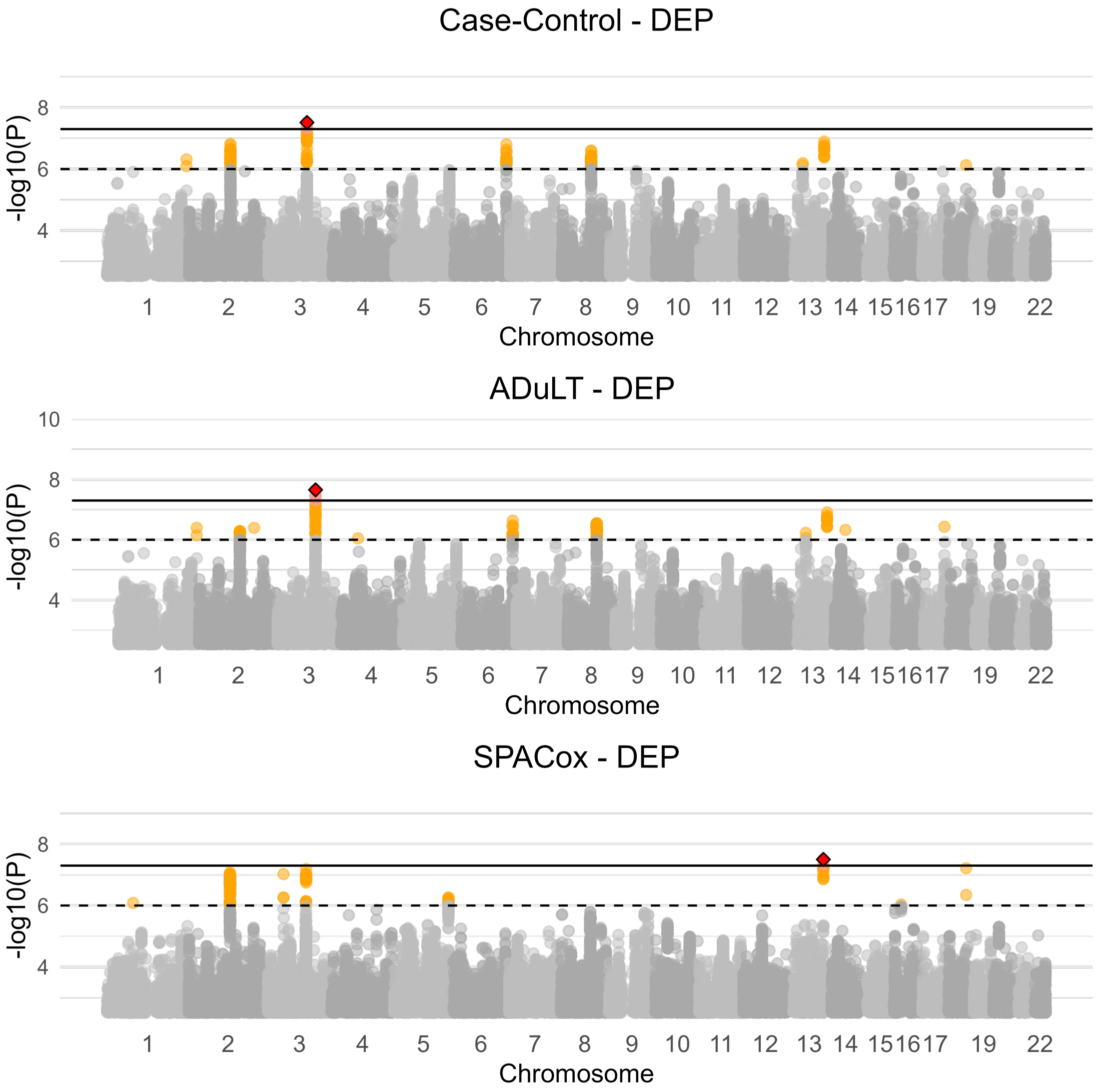

Supplementary Figure 27: **Manhattan plots for ADuLT, case-control status, and SPA-Cox of depression with age as a covariate for all phenotypes.** Manhattan plots for depression using the three methods. The orange dots indicate suggestive SNPs with a p-value threshold of 5×10^{-6}. The red dots correspond to Bonferroni-adjusted genome-wide significant SNPs with a p-value threshold of 5×10^{-8}. The diamonds correspond to the lowest p-value LD clumped SNP in a 500k base pair window with an $r^2 = 0.1$ threshold. All tests performed are two-sided.

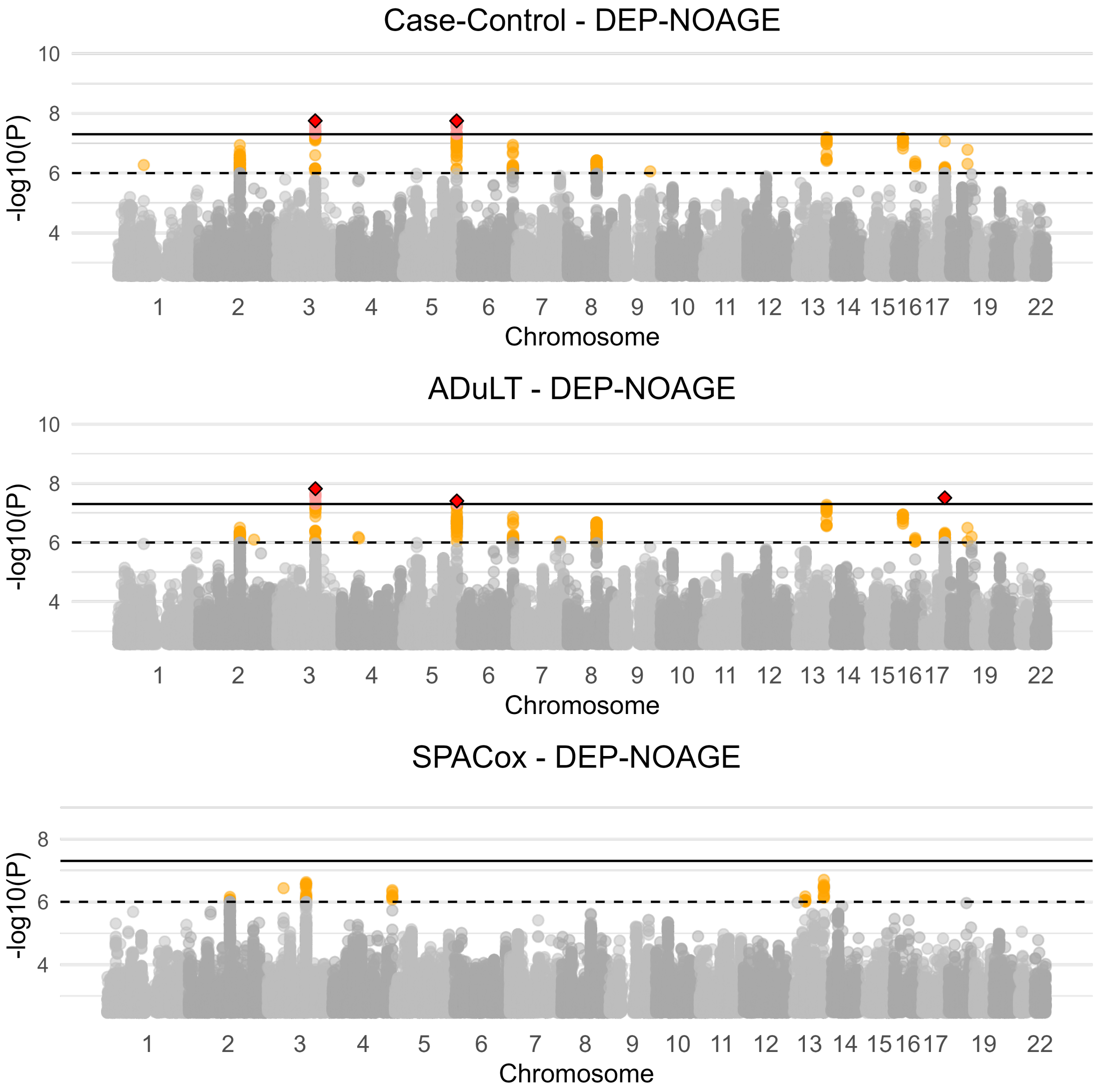

Supplementary Figure 28: **Manhattan plots for ADuLT, case-control status, and SPA-Cox of depression without age as a covariate for all phenotypes.** Manhattan plots for depression using all three methods. The orange dots indicate suggestive SNPs with a p-value threshold of 5×10^{-6}. The red dots correspond to Bonferroni-adjusted genome-wide significant SNPs with a p-value threshold of 5×10^{-8}. The diamonds correspond to the lowest p-value LD clumped SNP in a 500k base pair window with an $r^2 = 0.1$ threshold. All tests performed are two-sided.

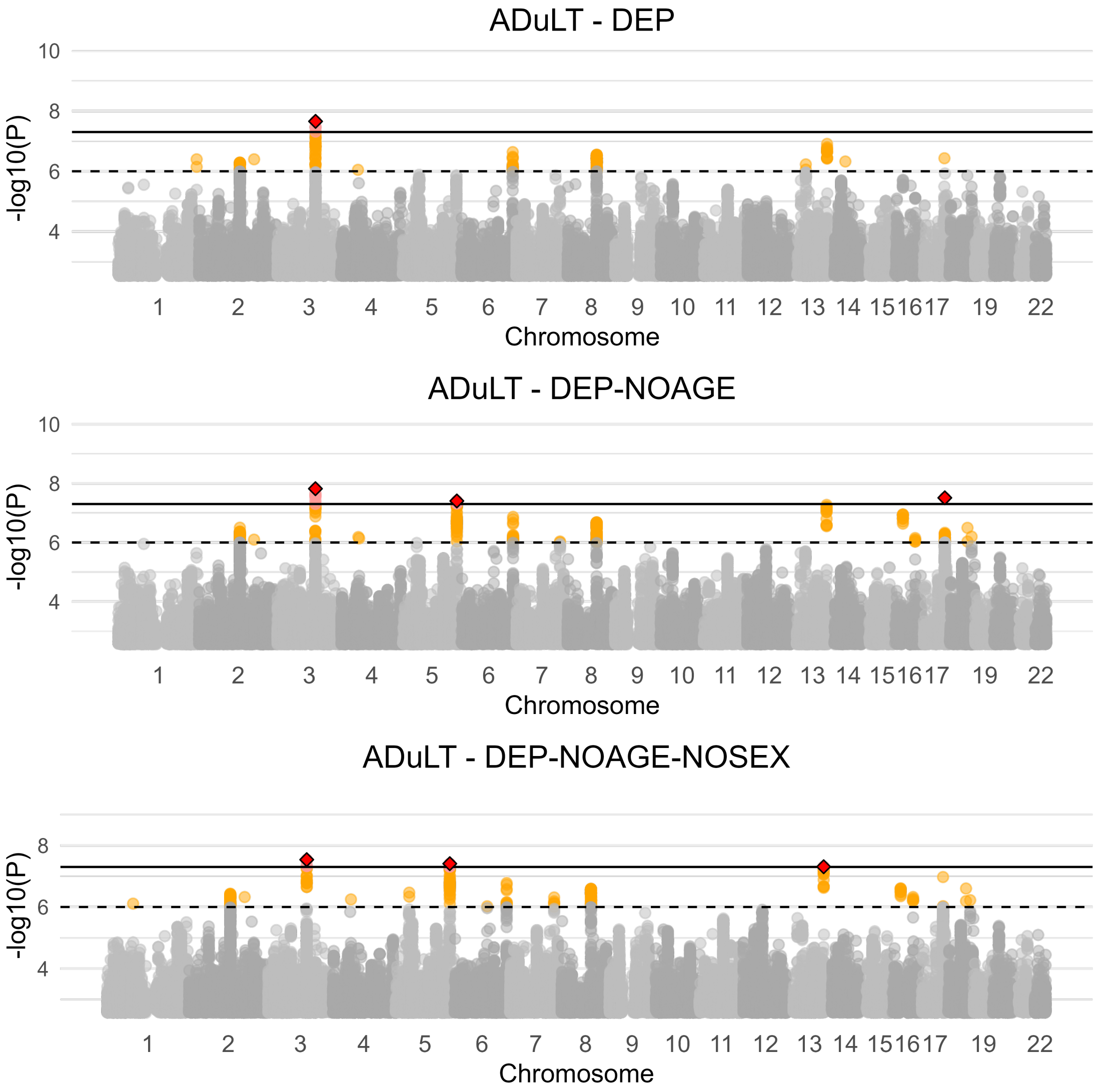

Supplementary Figure 29: **Manhattan plots for ADuLT with different included covariates.** Manhattan plots for depression using the ADuLT phenotype. The Manhattan plots are with age and sex as covariates, without age, and without both sex and age. The orange dots indicate suggestive SNPs with a p-value threshold of 5×10^{-6}. The red dots correspond to Bonferroni-adjusted genome-wide significant SNPs with a p-value threshold of 5×10^{-8}. The diamonds correspond to the lowest p-value LD clumped SNP in a 500k base pair window with an $r^2 = 0.1$ threshold. All tests performed are two-sided.

Schizophrenia

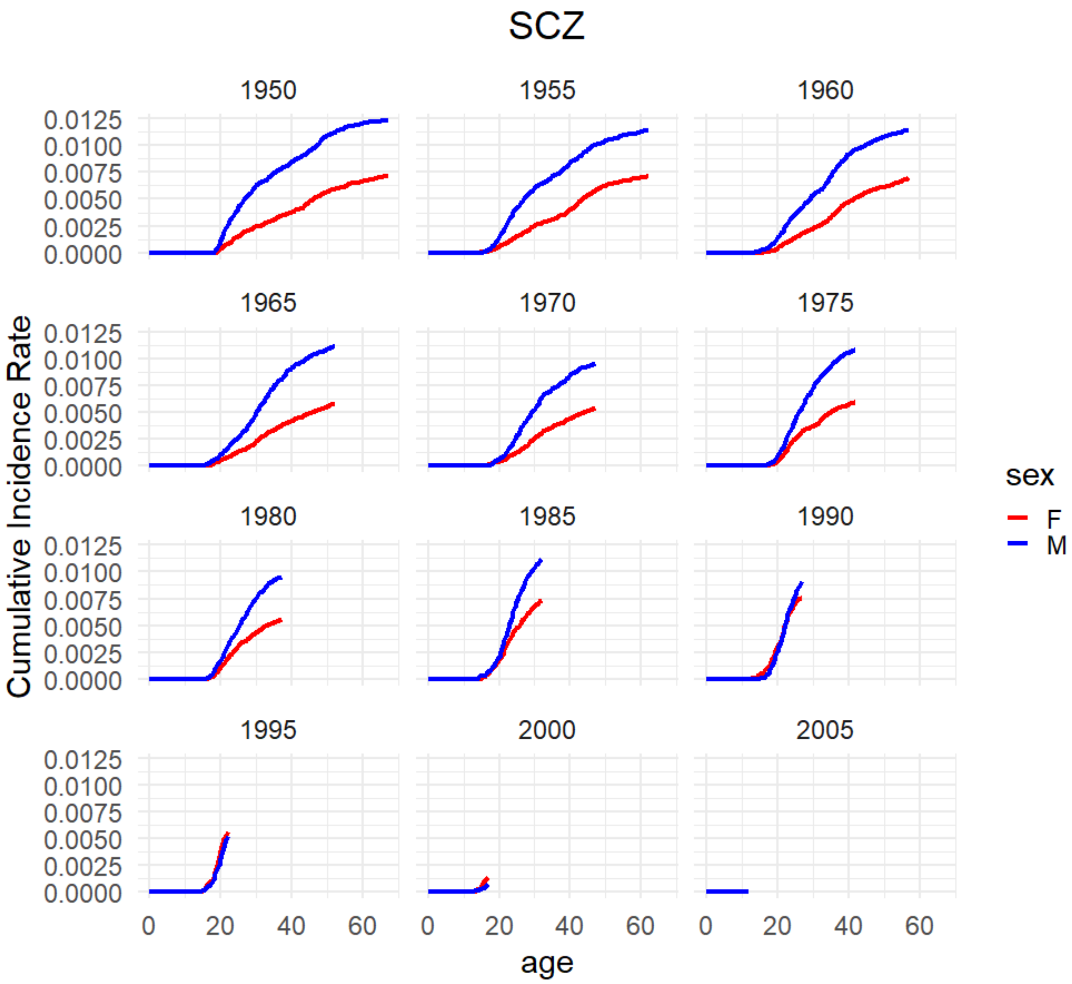

Supplementary Figure 30: **Cumulative incidence rates for Schizophrenia.** Cumulative incidence rates for schizophrenia in the Danish registers. The cumulative incidence proportions are stratified by birth year and sex.

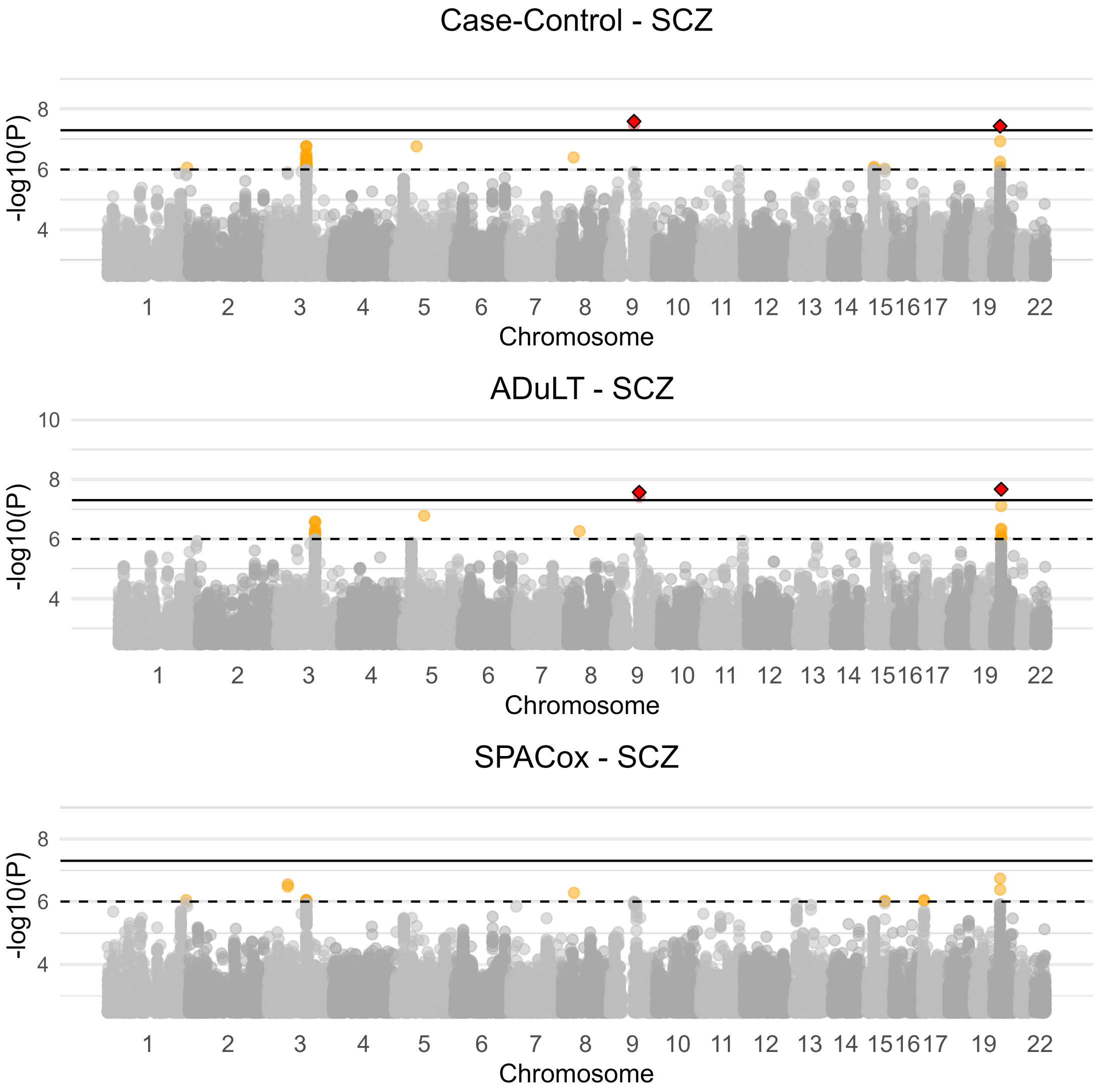

Supplementary Figure 31: **Manhattan plots for ADuLT, case-control status, and SPA-Cox of schizophrenia with age as a covariate for all phenotypes.** Manhattan plots for schizophrenia using the three methods. The orange dots indicate suggestive SNPs with a p-value threshold of 5×10^{-6}. The red dots correspond to Bonferroni-adjusted genome-wide significant SNPs with a p-value threshold of 5×10^{-8}. The diamonds correspond to the lowest p-value LD clumped SNP in a 500k base pair window with an $r^2 = 0.1$ threshold. All tests performed are two-sided.

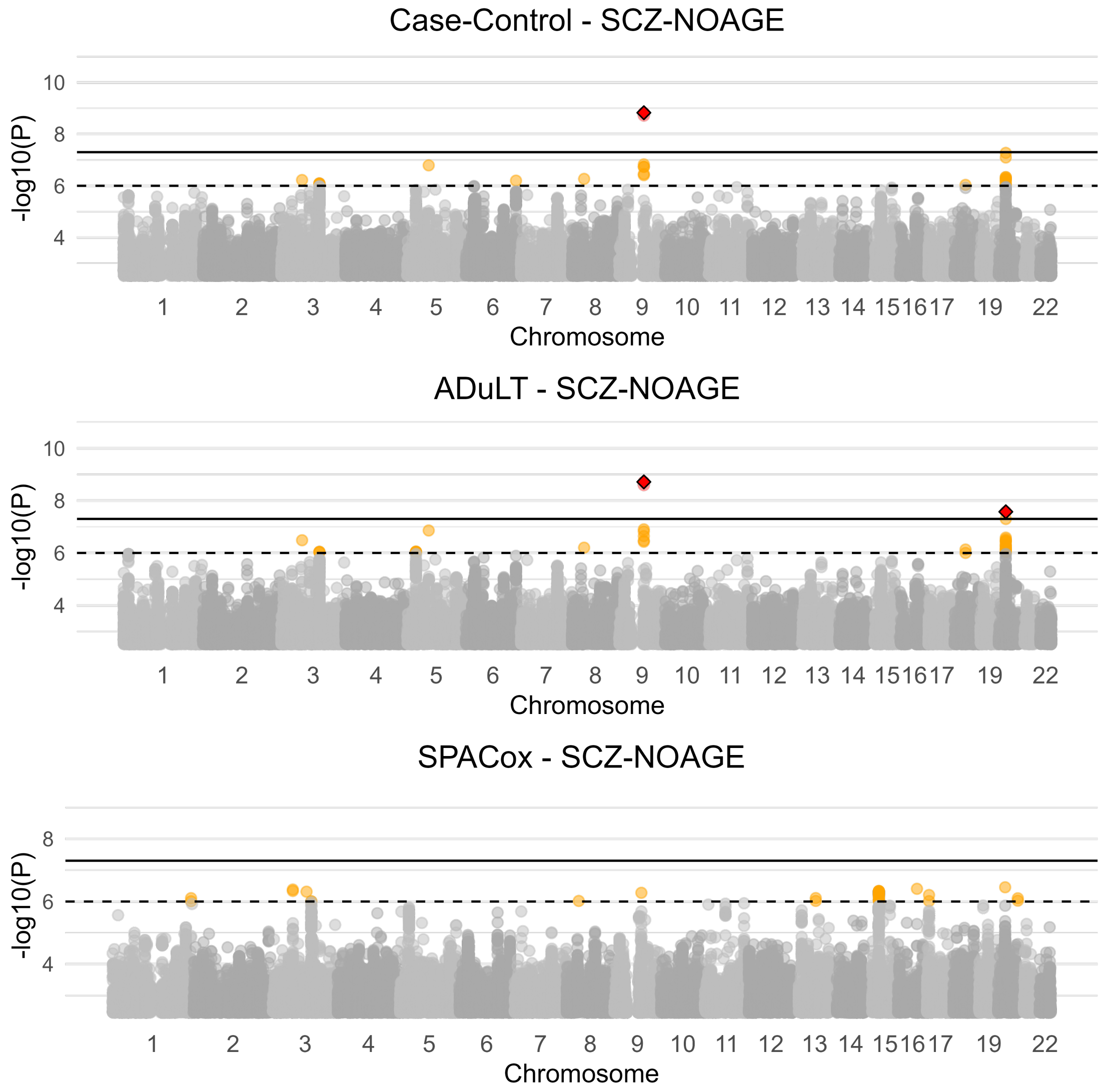

Supplementary Figure 32: **Manhattan plots for ADuLT, case-control status, and SPACox of schizophrenia without age as a covariate for all phenotypes.** Manhattan plots for schizophrenia using the three methods. The orange dots indicate suggestive SNPs with a p-value threshold of 5×10^{-6}. The red dots correspond to Bonferroni-adjusted genome-wide significant SNPs with a p-value threshold of 5×10^{-8}. The diamonds correspond to the lowest p-value LD clumped SNP in a 500k base pair window with an $r^2 = 0.1$ threshold. All tests performed are two-sided.

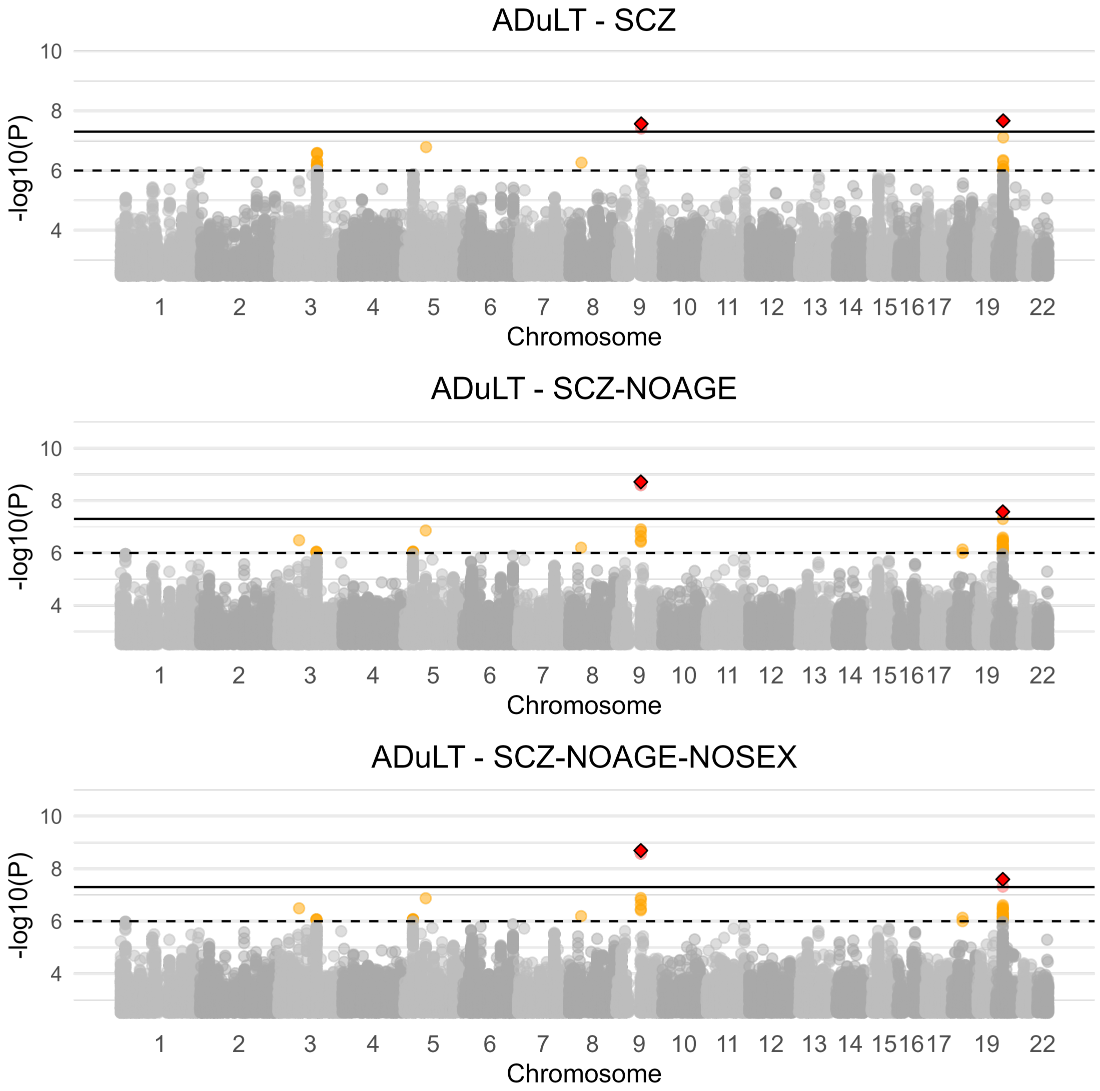

Supplementary Figure 33: **Manhattan plots for ADuLT with different included covariates.** Manhattan plots for schizophrenia using the ADuLT phenotype. The Manhattan plots are with age and sex as covariates, without age, and without both sex and age. The orange dots indicate suggestive SNPs with a p-value threshold of 5×10^{-6}. The red dots correspond to Bonferroni-adjusted genome-wide significant SNPs with a p-value threshold of 5×10^{-8}. The diamonds correspond to the lowest p-value LD clumped SNP in a 500k base pair window with an $r^2 = 0.1$ threshold. All tests performed are two-sided.

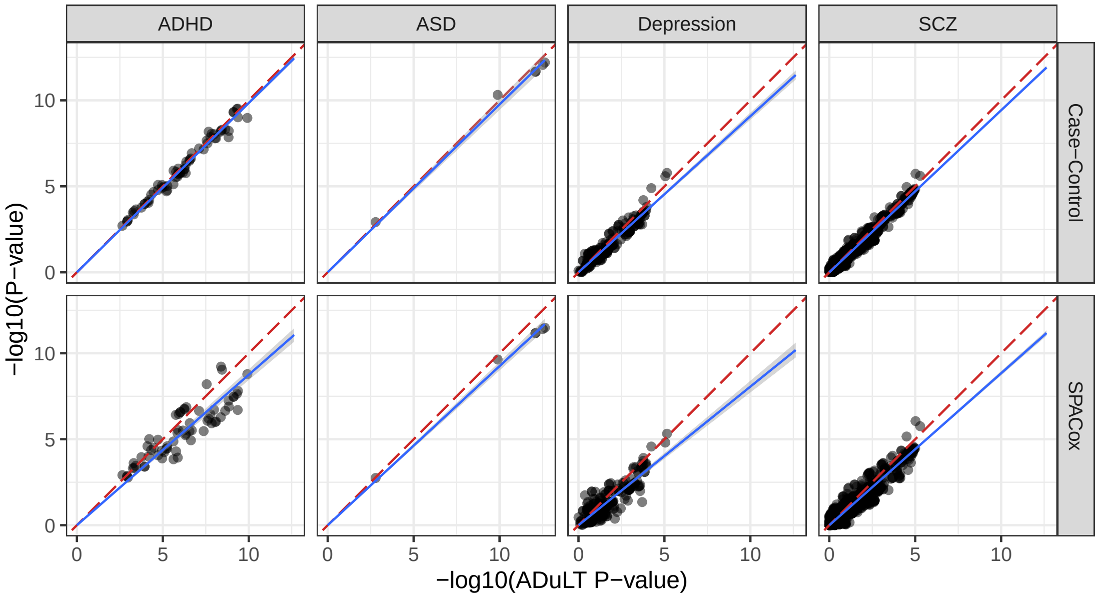

Supplementary Figure 34: **Validation of p-values in largest available meta-analysed PGC summary statistics.** The largest available meta-analysed PGC sumstats have been LD-clumped in the iPSYCH data (see methods and results for details) and the p-values for ADuLT without age as a covariate have been plotted against the case-control and SPACox p-values. The red dashed line indicate the identity line and the blue line indicate the best linear fit. The best linear line indicates that the ADuLT p-values are slightly higher on the externally identified SNPs.

Supplementary Tables

	ADHD	Autism	Depression	Schizophrenia
Control	36548	36741	36368	36921
Case	21738	18235	27507	11602

Supplementary Table 1: **Table including the number of individuals used for each iPSYCH disorders.** The number of cases and controls used in the GWAS for each iPSYCH phenotype. Numbers are shown after filtering for relatedness and restricting to a group of individuals with European ancestry.

Method	with age	with sex	total significant SNPs
ADuLT	no	no	20
	no	yes	20
	yes	yes	17
CaseControl	no	no	16
	no	yes	16
	yes	yes	17
SPACox	no	yes	8
	yes	yes	14

Supplementary Table 2: **Table including the sum of genome-wide significant associations across the four iPSYCH disorders.**

Variant ID	Chromosome: Position (hg38)	Effect size (SE)	ADuLT p-value (-log10(P))	Nearest gene	Selected previously reported associations
rs11210887	1:44076019	0.0331(0.0049)	10.8	PTPRF	smoking initiation, educational attainment[2,3]
rs11210887	1:44076019	0.0331(0.0049)	10.8	PTPRF	smoking initiation, educational attainment[2,3]
rs4660756	1:44383914	0.0284(0.0051)	7.48	ST3GAL3	educational attainment, ADHD[4,5]
rs7563362	2:620297	-0.0361(0.0065)	7.47	LINC01875, TMEM18	Type 2 diabetes, BMI[6,7]
rs4916723	5:87854395	-0.0359(0.0047)	13.7	LINC00461	ADHD, Educational Attainment, BMI[2,5,8]
rs12705966	7:114248851	0.0334(0.0052)	9.86	-	-
rs13236619	7:157827565	-0.0263(0.0046)	8.05	-	-
rs72673548	8:93292844	-0.0423(0.0076)	7.55	-	-
rs12346733	9:86727865	-0.0256(0.0047)	7.35	-	-
rs57806515	11:28628549	0.0282(0.0047)	8.87	-	-
rs704061	12:89771903	-0.0252(0.0046)	7.49	DUSP6, POC1B	ADHD, Educational Attainment, BMI[2,5,9]
rs4261436	14:33299482	-0.0257(0.0045)	7.86	AKAP6	Educational attainment, BMI, Type 2 diabetes[2,8,10]
rs4813421	20:21258053	0.0305(0.005)	8.92	-	-

Supplementary Table 3: **LD clumped Bonferroni-adjusted genome-wide significant SNPs based on the ADuLT phenotype for ADHD in iPSYCH.** All SNPs have a p-value below 5×10^{-8} and are ordered by chromosome and location. The table contains the rsID, chromosome, location (bp), effect size, standard error, and p-value for each SNP. Information on the closest gene and some selected previous associations for each SNP are included as well. All tests performed are two-sided.

Variant ID	Chromosome: Position (hg38)	Effect size (SE)	ADuLT p-value (-log10(P))	Nearest gene	Selected previously reported associations
rs8085882	18:22743899	0.0156(0.0029)	7.30	ZNF521	education attainment, smoking initiation[2,3]

Supplementary Table 4: **LD clumped Bonferroni-adjusted genome-wide significant SNPs for ADHD that are unique to Case-Control status in iPSYCH.** All SNPs have a p-value below 5×10^{-8}. The table contains the rsID, chromosome, location (bp), effect size, standard error, and p-value for each SNP. Information on the closest gene and some selected previous associations for each SNP are also included. All tests performed are two-sided.

Supplementary References

1. Therneau, T. M. *A Package for Survival Analysis in R* R package version 3.4-0 (2022). `https://CRAN.R-project.org/package=survival`.
2. Lee, J. J. *et al.* Gene discovery and polygenic prediction from a genome-wide association study of educational attainment in 1.1 million individuals. *Nat. Genet.* **50** (July 2018).
3. Liu, M. *et al.* Association studies of up to 1.2 million individuals yield new insights into the genetic etiology of tobacco and alcohol use. *Nat. Genet.* **51** (Feb. 2019).
4. Okbay, A. *et al.* Genome-wide association study identifies 74 loci associated with educational attainment. *Nature* **533** (May 2016).
5. Wu, Y. *et al.* Multi-trait analysis for genome-wide association study of five psychiatric disorders. *Transl. Psychiatry* **10** (June 2020).
6. Thorleifsson, G. *et al.* Genome-wide association yields new sequence variants at seven loci that associate with measures of obesity. *Nat. Genet.* **41** (Jan. 2009).
7. Vujkovic, M. *et al.* Discovery of 318 new risk loci for type 2 diabetes and related vascular outcomes among 1.4 million participants in a multi-ancestry meta-analysis. *Nat. Genet.* **52** (July 2020).
8. Zhu, Z. *et al.* Shared genetic and experimental links between obesity-related traits and asthma subtypes in UK Biobank. *J. Allergy Clin. Immunol.* **145** (Feb. 2020).
9. Pulit, S. L. *et al.* Meta-analysis of genome-wide association studies for body fat distribution in 694 649 individuals of European ancestry. *Hum. Mol. Genet.* **28** (Jan. 2019).
10. Mahajan, A. *et al.* Fine-mapping type 2 diabetes loci to single-variant resolution using high-density imputation and islet-specific epigenome maps. *Nat. Genet.* **50** (Nov. 2018).
