# Supplementary material for: ADuLT: An efficient and robust time-to-event GWAS
Source: Nat Commun. 2023 Sep 9;14:5553. doi: 10.1038/s41467-023-41210-z (PMC10492844; doi:10.1038/s41467-023-41210-z)
Supplement: Supplementary file 3 — Description of Additional Supplementary Files [file 41467_2023_41210_MOESM3_ESM.pdf]

Description of Additional Supplementary Files

File Name: Supplementary Data 1
Description: Excel file with summary information from the simulations, containing information such as power, average of the χ^2-statistics of the null SNPs, false positive rates for varying significance levels, etc. All summary information from the simulations has been combined into this Excel file and it contains several sheets. We explain what information each sheet holds and what the important columns are called. The first sheet is the raw simulation results for each replication (`v`). This includes information on power (`power`), number of false positives (`FP`) and causal SNPs (`causal`) identified at 5×10^{-8}. The average χ^2-statistics are also provided for the null SNPs (`mean_null_chisq`) and causal SNPs (`mean_causal_chisq`). This information is given for each choice of generative model (`gen_mod`), prevalence in the full data (`prev`), model used to assign phenotype under the generative model(`method`), total number of causal SNPs (`C`), and whether case ascertainment was present or not (`downsampling`). Each simulation setup has 10 replications, and information is available for each iteration. The second sheet provide averages across replications for a set of parameters. The average power (`avg_power`) and null χ^2-statistic (`avg_null`), as well as their standard error (`avg_power_se` and (`avg_null_se`) and number of replications that did not identify any of the causal SNPs (`no_causal`) is reported. The third sheet provide the run-times plotted in Figure 3. The number of individuals (`N`), SNPs (`M`), and cores used (`ncores`) are reported. The average run times (`mean_times`) and the standard error (`se_times`) are reported. The fourth sheet provide false positive rates (`FP_prop`) and standard errors (`FP_sem`) for a significance thresholds ranging (`alpha_lvl`) from 0.05, 0.005, to 5×10^{-8}. The number of false positives at a given significance threshold is given by (`FP`).
