# Supplementary material for: ADuLT: An efficient and robust time-to-event GWAS
Source: Nat Commun. 2023 Sep 9;14:5553. doi: 10.1038/s41467-023-41210-z (PMC10492844; doi:10.1038/s41467-023-41210-z)
Supplement: Supplementary file 5 — Reporting Summary [file 41467_2023_41210_MOESM5_ESM.pdf]

nature portfolio

Corresponding author(s): Emil Michael Pedersen (emp.ncrr@au.dk)

Last updated by author(s): Aug 8, 2023

Reporting Summary

Nature Portfolio wishes to improve the reproducibility of the work that we publish. This form provides structure for consistency and transparency in reporting. For further information on Nature Portfolio policies, see our Editorial Policies and the Editorial Policy Checklist.

Statistics

For all statistical analyses, confirm that the following items are present in the figure legend, table legend, main text, or Methods section.

n/a	Confirmed	
[ ]	[x]	The exact sample size (*n*) for each experimental group/condition, given as a discrete number and unit of measurement
[ ]	[x]	A statement on whether measurements were taken from distinct samples or whether the same sample was measured repeatedly
[ ]	[x]	The statistical test(s) used AND whether they are one- or two-sided<br>*Only common tests should be described solely by name; describe more complex techniques in the Methods section.*
[x]	[ ]	A description of all covariates tested
[ ]	[x]	A description of any assumptions or corrections, such as tests of normality and adjustment for multiple comparisons
[ ]	[x]	A full description of the statistical parameters including central tendency (e.g. means) or other basic estimates (e.g. regression coefficient) AND variation (e.g. standard deviation) or associated estimates of uncertainty (e.g. confidence intervals)
[ ]	[x]	For null hypothesis testing, the test statistic (e.g. *F*, *t*, *r*) with confidence intervals, effect sizes, degrees of freedom and *P* value noted<br>*Give P values as exact values whenever suitable.*
[x]	[ ]	For Bayesian analysis, information on the choice of priors and Markov chain Monte Carlo settings
[x]	[ ]	For hierarchical and complex designs, identification of the appropriate level for tests and full reporting of outcomes
[x]	[ ]	Estimates of effect sizes (e.g. Cohen's *d*, Pearson's *r*), indicating how they were calculated

Our web collection on statistics for biologists contains articles on many of the points above.

Software and code

Policy information about availability of computer code

Data collection	We did not collect any data for the paper. All information used has already been collected and described else where, e.g. the iPSYCH cohort. We did use a HPC cluster to reduce the computation time for simulations, real-world data, as well as offer the necessary data security needed for storage and analysis of genetic data. We used genomeDK (https://genome.au.dk)
Data analysis	We used R for all analysis. Most of the code used is based on the tidyverse packages. Notably, we used some custom R packages too. We used an implementation of SPACox that was provided by the author's of the meothd (https://githuv.com/wenjianBI/SPACox). Linear regression was performed with the bigsnpr package (https://github.com/privefl/bigsnpr). An implementation of ADuLT, which has previously been used for the method LT-FH++, can be found on github (https://github.com/EmilMiP/LTFHPlus). The code used for simulations and real-world analysis can also be found on github (https://github.com/EmilMiP/ADuLTCode).

For manuscripts utilizing custom algorithms or software that are central to the research but not yet described in published literature, software must be made available to editors and reviewers. We strongly encourage code deposition in a community repository (e.g. GitHub). See the Nature Portfolio guidelines for submitting code & software for further information.

Data

Policy information about availability of data

All manuscripts must include a data availability statement. This statement should provide the following information, where applicable:

- Accession codes, unique identifiers, or web links for publicly available datasets
- A description of any restrictions on data availability
- For clinical datasets or third party data, please ensure that the statement adheres to our policy

iPSYCH is approved by the Danish Scientific Ethics Committee, the Danish Health Data Authority, the Danish Data Protection Agency, Statistics Denmark, and the Danish Neonatal Screening Biobank Steering Committee. Owing to the sensitive nature of the iPSYCH data, individual level data can only be accessed through secure servers where downloading individual level information is prohibited. Each scientific project must be approved before initiation, and approval is granted to a specific Danish research institution. International researchers may gain data access through collaboration with a Danish research institution. More information about getting access to the iPSYCH data can be obtained at \url{https://ipsych.dk/en/about-ipsych}.

Research involving human participants, their data, or biological material

Policy information about studies with human participants or human data. See also policy information about sex, gender (identity/presentation), and sexual orientation and race, ethnicity and racism.

Reporting on sex and gender	We did not sample individuals for this analysis. We used the iPSYCH2015 sample. Details on the population can be seen from the preprint (https://doi.org/10.1101/2020.11.30.20237768)
Reporting on race, ethnicity, or other socially relevant groupings	We did not sample individuals for this analysis. We used the iPSYCH2015 sample. Details on the population can be seen from the preprint (https://doi.org/10.1101/2020.11.30.20237768)
Population characteristics	We did not sample individuals for this analysis. We used the iPSYCH2015 sample. Details on the population can be seen from the preprint (https://doi.org/10.1101/2020.11.30.20237768)
Recruitment	We did not sample individuals for this analysis. We used the iPSYCH2015 sample. Details on the population can be seen from the preprint (https://doi.org/10.1101/2020.11.30.20237768)
Ethics oversight	We did not sample individuals for this analysis. We used the iPSYCH2015 sample. Details on the population can be seen from the preprint (https://doi.org/10.1101/2020.11.30.20237768)

Note that full information on the approval of the study protocol must also be provided in the manuscript.

Field-specific reporting

Please select the one below that is the best fit for your research. If you are not sure, read the appropriate sections before making your selection.

☒ Life sciences ☐ Behavioural & social sciences ☐ Ecological, evolutionary & environmental sciences

For a reference copy of the document with all sections, see nature.com/documents/nr-reporting-summary-flat.pdf

Life sciences study design

All studies must disclose on these points even when the disclosure is negative.

Sample size	No sample size calculations were performed. The sample size is the result of selecting all eligible individuals with the disorder of interest, as well as a population representative control group (50k individuals).
Data exclusions	For the GWAS, filtering on relatedness and ancestry was performed, as relatedness is known to bias the analysis. Subsequent analysis was performed on a genetically homogeneous and largely unrelated set of individuals.
Replication	The simulations can be replicated with the provided code, if need be. The real world data analysis has not been replicated, since we do not have access to a suitable independent data set.
Randomization	The study is based on the iPSYCH cohort. The controls are a random sample of the population, while the cases consists of all individuals in the population that experienced a given disorder.
Blinding	We did not collect the data. We used the iPSYCH2015 sample. Details on the population can be seen from the preprint (https://doi.org/10.1101/2020.11.30.20237768)

Reporting for specific materials, systems and methods

We require information from authors about some types of materials, experimental systems and methods used in many studies. Here, indicate whether each material, system or method listed is relevant to your study. If you are not sure if a list item applies to your research, read the appropriate section before selecting a response.

Materials & experimental systems

n/a	Involved in the study
☒	☐ Antibodies
☒	☐ Eukaryotic cell lines
☒	☐ Palaeontology and archaeology
☒	☐ Animals and other organisms
☒	☐ Clinical data
☒	☐ Dual use research of concern
☒	☐ Plants

Methods

n/a	Involved in the study
☒	☐ ChIP-seq
☒	☐ Flow cytometry
☒	☐ MRI-based neuroimaging
